# A three-dimensional RNA motif mediates directional trafficking of *Potato spindle tuber viroid* from epidermal to palisade mesophyll cells in *Nicotiana benthamiana*

**DOI:** 10.1371/journal.ppat.1008147

**Published:** 2019-10-23

**Authors:** Jian Wu, Neocles B. Leontis, Craig L. Zirbel, David M. Bisaro, Biao Ding

**Affiliations:** 1 Department of Molecular Genetics, Center for Applied Plant Sciences, Center for RNA Biology, Infectious Diseases Institute, and Graduate Program in Molecular, Cellular, and Developmental Biology, The Ohio State University, Columbus, Ohio, United States of America; 2 Department of Chemistry and Center for Biomolecular Sciences, Bowling Green State University, Bowling Green, Ohio, United States of America; 3 Department of Mathematics and Statistics, Bowling Green State University, Bowling Green, Ohio, United States of America; Agriculture and Agri-Food Canada, CANADA

## Abstract

*Potato spindle tuber viroid* (PSTVd) is a circular non-coding RNA of 359 nucleotides that replicates and spreads systemically in host plants, thus all functions required to establish an infection are mediated by sequence and structural elements in the genome. The PSTVd secondary structure contains 26 Watson-Crick base-paired stems and 27 loops. Most of the loops are believed to form three-dimensional (3D) structural motifs through non-Watson-Crick base pairing, base stacking, and other local interactions. Homology-based prediction using the JAR3D online program revealed that loop 27 (nucleotides 177–182) most likely forms a 3D structure similar to the loop of a conserved hairpin located in the 3' untranslated region of histone mRNAs in animal cells. This stem-loop, which is involved in 3'-end maturation, is not found in polyadenylated plant histone mRNAs. Mutagenesis showed that PSTVd genomes containing base substitutions in loop 27 predicted by JAR3D to disrupt the 3D structure were unable to replicate in *Nicotiana benthamiana* leaves following mechanical rub inoculation, with one exception: a U178G/U179G double mutant was replication-competent and able to spread within the upper epidermis of inoculated leaves, but was confined to this cell layer. Remarkably, direct delivery of the U178G/U179G mutant into the vascular system by needle puncture inoculation allowed it to spread systemically and enter mesophyll cells and epidermal cells of upper leaves. These findings highlight the importance of RNA 3D structure for PSTVd replication and intercellular trafficking and indicate that loop 27 is required for epidermal exit, but not epidermal entry or transit between other cell types. Thus, requirements for RNA trafficking between epidermal and underlying palisade mesophyll cells are unique and directional. Our findings further suggest that 3D structure and RNA-protein interactions constrain RNA sequence evolution, and validate JAR3D as a tool to predict RNA 3D structure.

## Author summary

*Potato spindle tuber viroid* (PSTVd) is a small, circular non-coding RNA that systemically infects host plants. All functions required for replication as well as cell-to-cell and systemic spread are mediated by the RNA genome, whose 2D structure consists of multiple base paired stems and loops. However, a wealth of evidence indicates that most loops in RNA molecules are structured by non-canonical base pairs and other interactions. Here, we show that PSTVd loop 27 forms a 3D structure that is essential for replication. Analysis of an exceptional mutant revealed that loop 27 is also required for transit from infected epidermal cells to underlying palisade mesophyll cells, but is not needed for transport in the reverse direction or into other cell types. Thus, loop 27 is necessary for epidermal exit but not entry, and requirements for transit between epidermal cells and palisade mesophyll cells are unique and directional. PSTVd moves between cells through specialized structures known as plasmodesmata. Combined with previous findings, these studies suggest that plasmodesmal gates interconnecting most and perhaps all plant cell types are unique, allowing precise regulation of RNA transport and the establishment of distinct cellular boundaries.

## Introduction

The ability of multicellular organisms to grow, develop, and respond to the environment requires efficient, targeted, and regulated cell-to-cell and systemic communication involving the exchange of both small molecules and macromolecules. Cell boundaries play critical roles in balancing information transfer and cell autonomy, in part by acting as barriers that either facilitate or prevent the exchange of specific nucleic acids and proteins. As viruses and sub-viral pathogens must subvert these barriers to achieve successful infection of their hosts, they serve as useful models to study pathways that mediate macromolecular transfer between cells [1, 2]. At present, however, molecular mechanisms that regulate trafficking of macromolecules are incompletely understood.

In plants, developmental, physiological, and defensive processes involve the spread of specific mRNAs and proteins from the cells in which they are produced into neighboring and distant cells [3]. For example, grafting experiments have revealed that potato tuber formation [4–6], and tomato leaf morphogenesis [7, 8], require long-distance transport of specific mRNAs through the phloem. Identification of multiple mRNAs [9–11], as well as microRNAs (miRNAs) and small interfering RNAs (siRNAs) [12–14], in phloem sap further suggests non-cell autonomous roles for many RNA species. Trafficking of siRNAs mediates local and systemic gene silencing through pathways such as RNA interference and RNA-directed DNA methylation [15–17], which are essential for antiviral defense [18–20]. In addition, natural inter-organism exchanges of RNA molecules have been observed between host and parasitic plants [21, 22], as well as fungal pathogens and their hosts [23, 24].

Viruses and viroids achieve systemic infection through cell-to-cell spread and long-distance transport through the phloem. Most plant viruses and all viroids have RNA genomes that provide useful tools to investigate RNA trafficking pathways [25–28]. Virus trafficking requires viral movement proteins. By contrast, viroids are non-coding RNAs and biological functions necessary to establish infection are accomplished by genomic sequence and structure elements that interact with appropriate host proteins. While significant progress has been made, the precise molecular mechanisms of viral and viroid spread remain elusive, in part because the properties of cellular boundaries are poorly defined. A better understanding of cell-to-cell and systemic RNA trafficking in plants will require in-depth characterization of cellular boundaries at multiple levels and between different cell types.

We are using *Potato spindle tuber viroid* (PSTVd) to investigate the roles of unique structural motifs in RNA trafficking. To date, several motifs that mediate passage across distinct cellular boundaries have been identified [29–33]. However, an element necessary for PSTVd trafficking between upper epidermal cells and immediately underlying palisade mesophyll cells, which represents an early step in systemic infection initiated by mechanical leaf abrasion (rub inoculation), has not been identified. Mechanical inoculation is a common PSTVd transmission mechanism both in the field and the laboratory.

**Fig 1:**
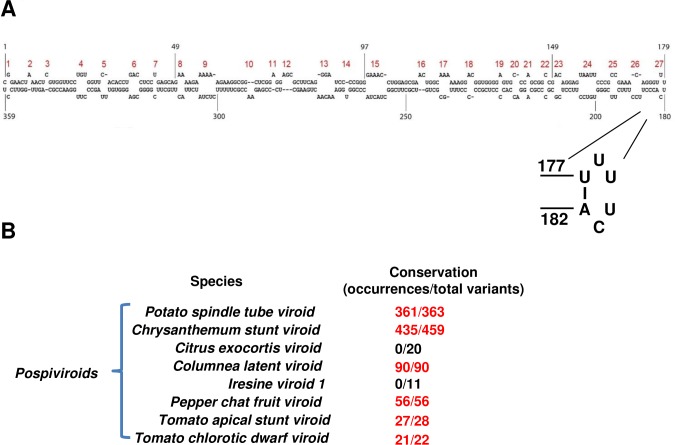
PSTVd secondary structure and conservation of the 5'-UUUUCA-3' stem-loop among *Pospiviroid* species. (A) Secondary structure of PSTVd. Nucleotide coordinates are indicated and loops/bulges are numbered 1 to 27 (red). The inset highlights loop 27 and the Watson-Crick U-A base pair that closes the loop. (B) Conservation of the 5'-UUUUCA-3' stem-loop across eight *Pospiviroid* species. The number of occurrences observed in the total number of variants analyzed for each species is indicated. Numbers in red indicate that the loop 27 secondary structure is present in a majority of variants.

The 359 nucleotide (nt) PSTVd RNA genome folds into a well-established secondary structure that contains 26 base-paired stems and 27 loops [27] (Fig 1A). In this study, a combination of genetic, molecular, and cellular approaches was used to characterize the three-dimensional (3D) structural features and functional roles of terminal loop 27 (nt 177–182) in replication and trafficking between cells. Loop 27 (5'-UUUUCA-3') is identical in sequence to the loop of a hairpin found in the 3' untranslated region (UTR) of histone mRNAs. The histone mRNA stem-loop is highly conserved among animal species but is not present in polyadenylated plant histone mRNAs. In animals, this element forms a distinct 3D structure that interacts with stem-loop binding protein (SLBP). SLBP promotes recruitment of U7 snRNP, which is required for endonucleolytic cleavage that matures the 3' end of histone mRNAs which lack poly(A) [34–36]. As the role of this RNA element in replication and trafficking of a plant infecting viroid was unclear, a mutagenesis-based functional characterization was conducted guided by the JAR3D (Java-based Alignment of RNA using 3D structure) online program to predict the structures of loop 27 mutants and to suggest compatible and disruptive mutations [37].

Our results indicate that the native 3D structure of loop 27 is required for PSTVd replication but not for trafficking between most cell types. However, it is essential for spread of viroid RNA from the upper epidermis to underlying palisade mesophyll cells, but not for transit in the opposite direction (i.e. from palisade mesophyll to epidermal cells). Thus, the requirements for RNA trafficking across the boundary between epidermal and palisade mesophyll cells are unique and directional. Our findings further suggest that RNA sequence evolution is constrained by 3D structure and RNA-protein interactions, and highlight the utility of the JAR3D prediction program as a tool to assess RNA 3D structure.

## Results

### The 5'-UUUUCA-3' stem-loop (loop 27) is conserved within the genus *Pospiviroids*

Using sequence data available from the National Center for Biotechnology Information (NCBI) (Ribovaria; https://www.ncbi.nlm.nih.gov/genomes/GenomesGroup.cgi?taxid=2559587) the genomic sequences of 363 PSTVd variants were obtained. Variant nucleotides were mapped to the established secondary structure of the PSTVd intermediate strain (PSTVd-I) (Fig 1A), and variant secondary structures were assessed using Mfold (http://unafold.rna.albany.edu/?q=mfold). From this analysis, the 5'-UUUUCA-3' stem-loop was found in 361 of 363 PSTVd variants (Fig 1B). Of the remaining two variants, one (NCBI ID: KX159282.1) had a loop 27 sequence that differed by one base (5'-CUUUCA-3'), while the other (NCBI ID: KX159281.1) differed at three bases (5'-AUUUUU-3'). Both variants also had additional mutations at other sites.

The more than 30 viroid species presently identified are distributed between two families. Members of the *Avsunviroidae* (type member *Avocado sunblotch viroid*) replicate in the chloroplast, while the *Pospiviroidae* (type member PSTVd) replicate and accumulate in the nucleus. Based largely on sequence homology, the *Pospiviroidae* is further divided into three subfamilies and five genera [38]. PSTVd is placed in the genus *Pospiviroids*, along with at least seven other species. To assess conservation of the hairpin loop among both viroid families, variant sequences from 32 classified species were surveyed. Variants were scanned for the 5'-UUUUCA-3' sequence, which when identified was mapped to established secondary structures and/or analyzed using Mfold to verify the stem-loop [39–41]. We found that, with the exception of *Citrus exocortis viroid* (CEVd) and *Iresine viroid* (IrVd), the 5'-UUUUCA-3' stem-loop is highly conserved within pospiviroids and their variants (Fig 1B), suggesting it is important for infectivity of these species. CEVd and IrVd have loops with related but different sequences (5'-GCUCGAC-3' and 5'-GCUCGUC-3', respectively) at a location analogous to PSTVd loop 27. The 5'-UUUUCA-3' sequence is not found outside the *Pospiviroids* (S1 Fig).

### Chemical probing validates the tertiary structure model for loop 27

As the loop 27 sequence is highly conserved across PSTVd variants, 5'-UUUUCA-3' was used as the query sequence for homology-based modeling using JAR3D (RNA 3D Motif Atlas version 1.18), an online program based on hybrid Stochastic Context-Free Grammars (SCFG) and Markov Random Fields (MRF) [37]. SCFG/MRF models are constructed using atomic-resolution RNA 3D structures, employing core principles of isostericity of non-Watson-Crick (WC) base pairing and other base interactions [42]. Similar 3D motifs can occur in nonhomologous RNAs or at different sites in the same RNA molecule, and recurrent motif geometries are more conserved than the sequences that comprise them. JAR3D searches an RNA structure database and provides a list of known 3D motifs that are compatible with a query sequence.

**Fig 2:**
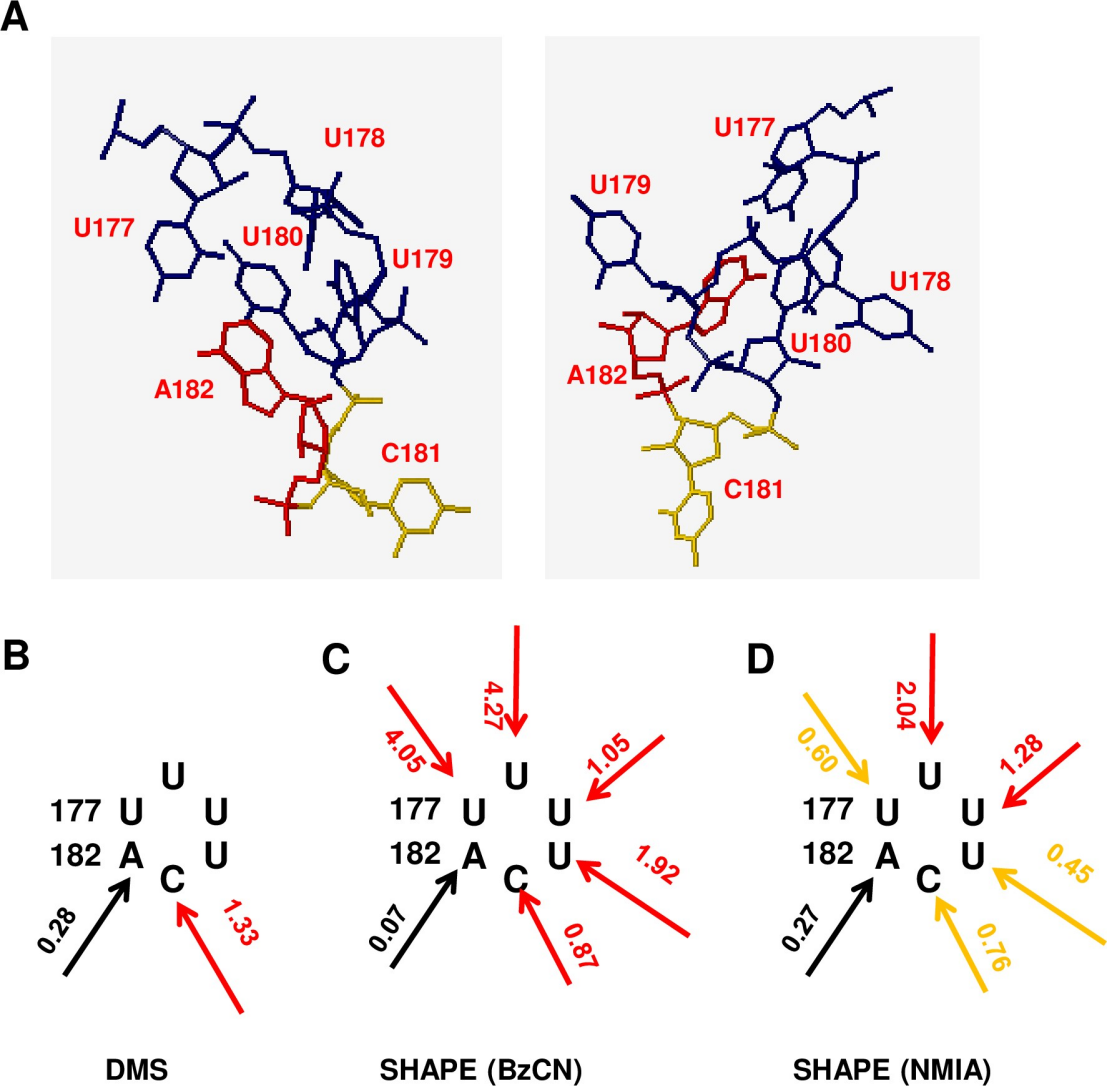
Tertiary structure model of loop 27 and validation by chemical modification. (A) Model of loop 27 obtained using the JAR3D program. Two different views are shown. The loop is closed by a Watson-Crick (WC) base pair between U177 and A182, and has two bases within the structure (U178 and U180). U180 stacks on the closing base pair. Two bases (U179 and C181) bulge out of the structure. (B) DMS modification. High reactivity of C181 (red arrow) indicates its WC edge is available for modification, whereas low reactivity (black arrow) indicates the WC edge of A182 is not. (C) SHAPE reactivity using BzCN. (D) SHAPE reactivity using NMIA. SHAPE modifies flexible (usually unpaired) bases. Low reactivity indicates a higher probability of base pairing. SHAPE reactivity is indicated by color: red = high (>0.85), orange = intermediate (0.4–0.85), black = low (0–0.40).

A search using the loop 27 sequence determined that the most promising model is Motif HL_27353.2 (UNCG-like) (http://rna.bgsu.edu/rna3dhub/motif/view/HL_27353.2), which contains seven RNA 3D structures from different RNA molecules resolved by either X-ray diffraction or cryo-electron microscopy (HL_4TV0_001, HL_4QIL_002, HL_4QI2_001, HL_4CUV_004, HL_4AQ7_002, HL_3U5H_004 and HL_1ZBH_001). Among the seven structures, the sequence of HL_4TV0_001 (Protein Data Bank ID: 4TV0) (http://rna.bgsu.edu/rna3dhub/loops/view/HL_4TV0_001; RNA-protein co-structure; click Show neighborhood to see interactions with protein) is identical to PSTVd loop 27. HL_4TV0_001 is part of the histone mRNA 3' UTR stem-loop (hereafter histone stem-loop) that interacts with SLBP. Based on HL_4TV0_001, a tertiary structure model of loop 27 was constructed. As shown in Fig 2A, the loop is closed by a WC base pair between U177 and A182. The loop region has two bases that bulge outside the structure (U179 and C181) and two bases within the structure (U178 and U180), with U180 stacked on the WC pair. There is no evidence for non-WC pairing within the loop, and in the absence of an interacting protein the loop nucleotides are likely disordered.

Chemical probing of PSTVd RNA was carried out to evaluate the structural model. Consistent with the model, dimethyl sulfate (DMS), which modifies exposed WC edges of unpaired A and C nucleotides, showed high reactivity for bulged out C181 and low reactivity at A182, which is base paired (Fig 2B). Further, selective 2'-hydroxyl acylation analyzed by primer extension (SHAPE), which is sensitive to the flexibility of RNA bases, showed that A182 had low reactivity to benzoyl cyanide (BzCN) consistent with base pairing, while loop bases displayed high reactivity (Fig 2C). (Primary SHAPE data for wild type loop 27 and selected loop 27 mutants is shown in S2 Fig) Similar results were obtained in previous DMS experiments and SHAPE studies performed using BzCN [43–45]. The high BzCN reactivity of U177 is somewhat surprising, as the model predicts that it forms a base pair with A182. However, it is possible that in the absence of factors that might stabilize the structure (such as a binding protein as is present in the HL_4TV0_001 model), A182 is also able to pair with U178, U179, and/or U180. Thus, in solution A182 may not always be paired with U177.

Depending on reaction times and other factors, different SHAPE probing chemicals can yield different results. BzCN reacts with a very fast half-life (250 milliseconds), whereas the half-life of N-methylisatoic anhydride (NMIA) is considerably longer (260 seconds) [46]. Upon probing loop 27 with NMIA, we observed that most bases were somewhat less reactive. However, U177 reactivity was considerably reduced from 4.05 observed with BzCN to 0.60 (Fig 2D). In another SHAPE study that employed NMIA and 2-methylnicotinic acid imidazolide (NAI) to probe PSTVd, U177 displayed low reactivity (less than 0.40) with both chemicals [47]. A comparison of SHAPE results obtained using BzCN, NMIA, or NAI for loop 27 and the flanking stem region is shown in S3 Fig. We concluded that the ability of SHAPE to discern the closing U177-A182 WC pair depends on the probing chemical employed, and that chemical probing largely validates the 3D model. The model is also consistent with RNase S1 probing, which showed that with the exception of U177 and A182, loop 27 bases are unpaired [43].

### Functional mutagenesis validates loop 27 model structure

Mutagenesis was carried out to assess the role of loop 27 in PSTVd infection. Further, as RNA 3D structure can determine function, mutagenesis could also validate the structural model. With this in mind, we designed a number of mutant sequences and used JAR3D to align them to the 5'-UUUUCA-3' model. JAR3D cutoff scores provided an estimate of the compatibility of a specific sequence with the predicted 3D model structure. The 15 PSTVd mutants tested spanned a range of cutoff scores (Table 1). For example, a U177A/A182U double mutant with a high cutoff score (88.69) was considered highly likely to form a loop 27 structure similar to the predicted model. Mutants with moderate scores (e.g. C181G, 46.68 and U179C, 28.42) were considered capable but perhaps less likely to form a similar structure. A mutation with a negative score (e.g. U179A/C181A, -26.29) was likely to disrupt the structure.

**Table 1:** Summary of PSTVd loop 27 mutant phenotypes.

Mutants	Cutoff score	Replication	Trafficking	Progeny Sequences	Predicted Impact
U179G	28.42	10/10	9/10	**U179G (5)**	Maintain structure and potential binding sites
U179C	28.42	10/10	10/10	**U179C (6)** U179C/C233U/C234U (1)	
C181G	46.68	10/10	10/10	**C181G (5)**	
U177A/A182U	88.69	10/10	10/10	**U177A/A182U (3)**	
U179A/C181A	-26.29	0/10	0/10	N/A	Disrupt structure/maintain potential binding sites
U179C/C181U	-9.35	0/10	0/10	N/A	
U177A	57.10	9/10	1/10	WT (3) ΔA89 (1)	Maintain structure/disrupt potential binding sites
U177C	65.29	0/10	0/10	N/A	
U178C	28.42	10/10	9/10	WT (4)	
U178G	28.42	10/10	8/10	WT (5)	
U180C	46.68	0/10	0/10	N/A	
A182G	67.82	10/10	1/10	WT (4)	
U177C/A182G	91.09	7/10	0/10	N/A	
U178G/U179G	‐32.98	10/10	0/10	N/A	Disrupt structure and potential binding sites
U180A/C181A	-19.59	0/10	0/10	N/A	

Loop 27 mutants tested are grouped according to predicted impact. JAR3D cutoff scores indicate the likelihood a mutant will conform to the predicted loop 27 structure. Higher scores are more compatible with the structural model. RNA blot analysis of extracts obtained from rub-inoculated leaves of 10 plants collected 10 dpi served as a replication assay, while analysis of extracts obtained 28 dpi from systemically infected leaves served as a trafficking assay. Infection rates (per 10 plants) are indicated. Full-length progeny genomes in pooled RNA samples from systemically infected leaves were cloned by RT-PCR and sequenced. Progeny sequences (number) retaining the mutation are indicated in bold. Other progeny either reverted to wild type (WT) and/or accumulated new mutations. Differences from wild type sequence are indicated. The atypical U178G/U179G mutant is highlighted in red.

**Fig 3:**
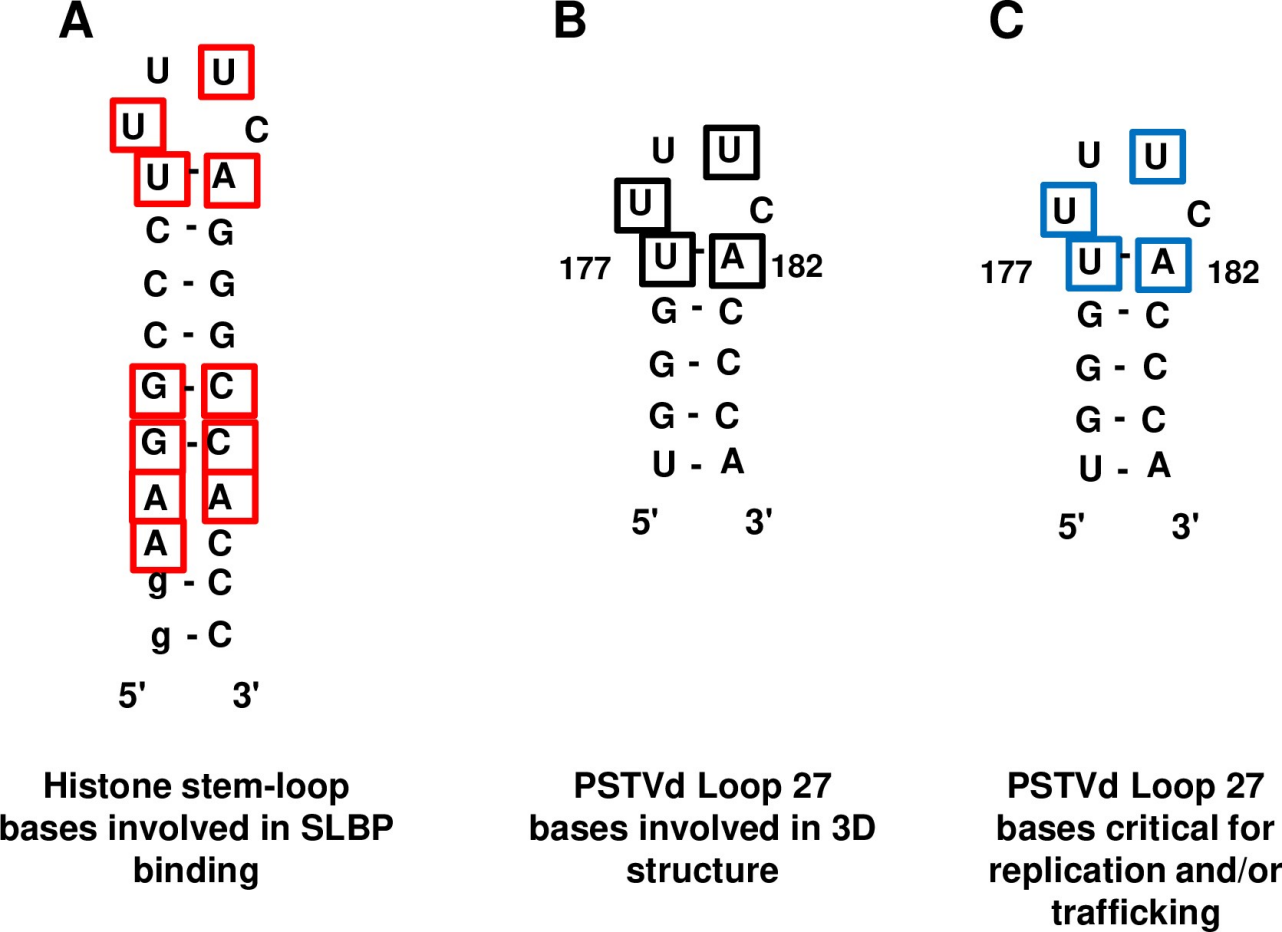
Comparison of conserved protein binding sites in the histone 3' UTR mRNA stem-loop and loop 27 bases critical for PSTVd replication and trafficking. (A) Residues in the histone stem-loop involved in SLBP binding are indicated by red boxes. Residues in lower case were added to stabilize the structure for *in vitro* binding assays. Exchanging the closing U-A pair with an A-U pair reduced but did not eliminate SLBP binding (Zanier et al., 2002) [35]. (B) Bases predicted to be involved in the PSTVd loop 27 3D structure and to contact an unknown plant binding protein(s) (black boxes). (C) Summary of loop 27 sites critical for replication and/or trafficking (blue boxes), as determined by single site mutagenesis. Note correlation between mutagenesis data and structure and binding site predictions.

We also considered the possibility that nucleotide sequence might be an important functional determinant. The histone stem-loop used to model loop 27 interacts with SLBP, and nucleotides conserved across different animal species are known to be important for SLBP binding [35] (Fig 3A). In PSTVd loop 27, these conserved residues correspond to U177 and A182 that close the loop, and U178 and U180 that participate in the 3D structure (Fig 3B). Additional bases important for SLBP binding lie in an extended stem that is absent from loop 27. Nevertheless, given the conserved structures of loop 27 and the loop of the histone hairpin, we speculated that an unknown plant protein (or proteins) might engage loop 27 using base contacts similar to those employed by SLBP (Fig 3A and 3B). Thus, mutants were classified into four groups based on predicted maintenance of structure and potential binding sites (Table 1).

Full-length PSTVd RNAs representing each of the mutants were rub-inoculated onto the upper surfaces of the first two true leaves of 10 *N*. *benthamiana* plants, and inoculated (local) leaves and upper non-inoculated (systemic) leaves were collected at 10- and 28-days post-inoculation (dpi), respectively. As PSTVd causes asymptomatic infections in this host, RNA preparations from harvested leaves were analyzed by blot hybridization to detect replication in inoculated leaves and systemic trafficking to upper leaves, and RNA blot signals were quantitated and compared relative to wild type controls present in each experiment. In inoculated leaves, circular progeny genomes are easily distinguished from linear inoculum due to their much slower mobility in polyacrylamide gels (S4 Fig). Full-length progeny genomes in pooled RNA samples extracted from systemic leaves, and in some cases inoculated local leaves, were recovered by RT-PCR, then cloned and sequenced to ascertain the genetic stability of mutations. RT-PCR was performed in such a way that only that only cDNA derived from circular progeny genomes would generate a detectable product (see Materials and Methods). The invariably high infection rate of wild type PSTVd in both local and systemic leaf assays (10/10 plants), and its inherent genetic stability, served as a positive control.

Only four of the 15 mutants were systemically infectious and genetically stable, and these were the only ones predicted to maintain both the 3D structure and potential protein binding sites (Table 1). Three of these (U179G, U179C, and C181G) affected bases that bulge out of the structure and one (double mutant U177A/A182U) exchanged the U-A pair that closes the loop for an A-U pair. By contrast, mutants predicted to disrupt the 3D structure but retain potential binding sites (double mutants U179A/C181A and U179C/C181U) failed to replicate in inoculated leaves.

Of six single mutants predicted to retain 3D structure but disrupt potential binding sites, four (U177A, U178C, U178G, and A182G) were able to infect local leaves, while two (U177C and U180C) were unable to replicate. However, the mutants capable of replication either failed to traffic systemically, or progeny genomes recovered from systemically infected leaves had reverted to the wild type (WT) sequence. The double mutant in this class (U177C/A182G) exchanged the closing U-A pair for a C-G pair. This mutant was capable of infecting inoculated leaves, albeit at a reduced rate, but was unable to spread systemically. As noted above, U177A/A182U was systemically infectious and stable. Thus, a U-A pair (wild type) or an A-U pair is allowed at nucleotides 177 and 182, but not a C-G pair.

Of the two double mutants predicted to disrupt both 3D structure and potential protein binding sites, U180A/C181A was not able to infect local leaves. Surprisingly, U178G/U179G was the only structurally incompatible mutant that was able to replicate in inoculated leaves, although it was not systemically infectious following rub inoculation. This exceptional mutant, which had the lowest cutoff score of all mutants tested (-32.98), was investigated further in experiments described below.

Quantitation of RNA blot signals was performed by averaging RNA blot signal density from the 10 plants inoculated with each mutant, and normalizing relative to averaged wild type PSTVd signals from 10 plants that served as a control for each experiment. Mutants that were able to replicate in inoculated (local) leaves could be placed in three groups: those that accumulated progeny at levels approaching or exceeding wild type (0.86–1.42; U177A, U179C, U177A/A182U, U177C/U182G), those that showed moderately reduced levels (0.60–0.68; U179G, U181G, U182G), and those with substantial reductions in progeny accumulation (0.26–0.36; U178C, U178G, and U178G/U179G) (S1 Table). The latter group share a mutation at U178 and, at least for U178G/U179G, the apparent reduction can be explained in part by its confinement to a single cell layer (see below). Quantitation of RNA blot signals from systemically infected leaves revealed that three of the four systemically infectious mutants accumulated to wild type or near wild type levels in systemic leaves (0.86–1.11; U179G, U179C, U177A/A182U), and the exception (0.66; C181G) showed moderately reduced accumulation (S1 Table).

In these studies, infection of local rub-inoculated leaves served as a surrogate replication assay. By this criterion, five mutants (U179A/C181A, U179C/C181U, U180A/C181A, U177C, and U180C) failed to replicate. Three of these mutants have negative cutoff scores, while two have positive scores. To rule out the possibility that absence of signal in RNA blots was due to an inability to spread cell-to-cell, all five mutants were subsequently tested for replication in *N*. *benthamiana* protoplasts. RNA blot analysis suggested that all of the mutants could replicate in transfected protoplasts, however, progeny genome sequencing revealed that all had reverted to wild type and accumulated additional mutations (S5 Fig, S2 Table). We concluded that the tested mutants had indeed lost the ability to replicate. We also wondered whether mutants that were replication-competent but reverted to wild type upon systemic spread were genetically stable in inoculated local leaves. While isolating and sequencing progeny clones from inoculated leaves is technically challenging, five were obtained and sequenced for two selected mutants. We found that four of five U178C progeny retained the mutation and one reverted to wild type, while three of five U178G progeny maintained the mutation and two were wild type. Thus, both mutants were capable of replication in inoculated leaves, although revertants responsible for subsequent systemic spread were also detected. On the basis of these studies, we concluded that local leaf inoculation is a valid replication assay.

In summary, of the eleven mutants predicted to be compatible with the 3D model structure for loop 27, only two (U177C and U180C) were unable to replicate in local leaves. Of the four mutants predicted to disrupt the structure, only one was able to replicate in inoculated leaves (again, the U178G/U179G mutant is discussed further below). Thus, it appears that a loop 27 structure similar to the histone stem-loop is necessary but not sufficient to form a replication-competent PSTVd genome. Requirements for successful replication and systemic trafficking are clearly more stringent, as only mutants predicted to maintain both structure and potential protein binding sites (U179G, U179C, C181G, and U177A/A182U) were systemically infectious. Interestingly, these mutations impact bases predicted to bulge out of the structure or exchange the closing U-A pair for an A-U pair. Thus, there is strong correlation between mutagenesis results and structural and binding site predictions (Fig 3C).

### Loop 27 is required for transport of PSTVd out of epidermal cells

**Fig 4:**
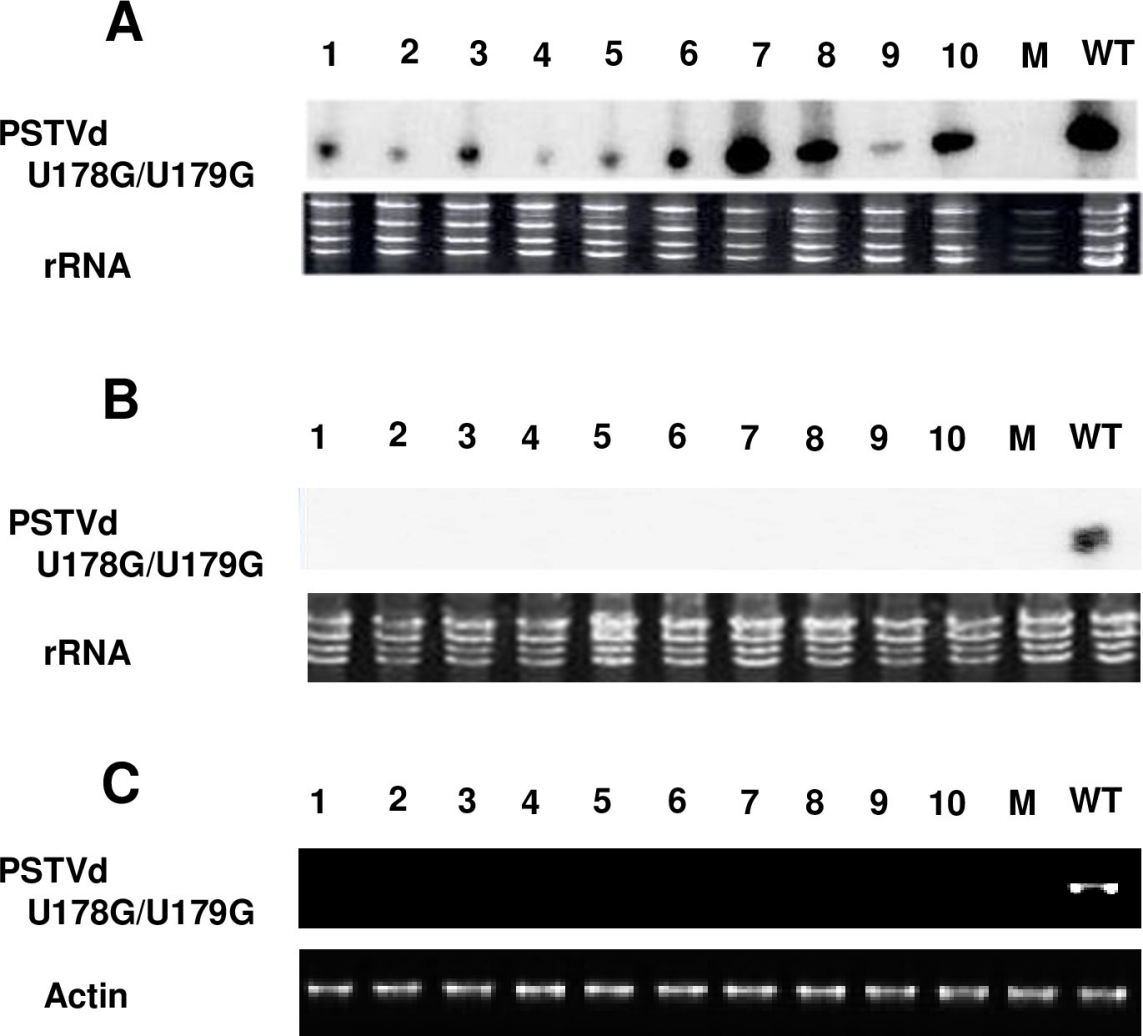
U178G/U179G replicates but fails to exit local leaves following rub-inoculation. Total RNA was collected from: (A) inoculated leaves, (B) upper systemically infected leaves, or (C) petioles of inoculated leaves of 10 plants inoculated with U178G/U179G or wild type PSTVd (WT, one plant, positive control). Mock inoculation (M) was a negative control. (A) RNA blot assay indicates U178G/U179G replication in rub-inoculated leaves. (B) RNA blot assay indicates U178G/U179G is unable to traffic to upper leaves following rub inoculation. (C) RT-PCR indicates U178G/U179G is not present in petioles and fails to exit inoculated leaves. In A and B, the region of the blot corresponding to circular progeny genomes is shown. Loading controls were ribosomal RNA (rRNA) (A and B) and RT-PCR of actin mRNA (C), detected by ethidium bromide staining. Images are representative of 10 (A and B) and three (C) independent experiments.

As noted above, the U178G/U179G mutation was predicted to disrupt both the 3D structure of loop 27 as well as potential binding sites. Yet, although systemic spread did not occur, RNA blot analysis indicated a 100% infection rate for this mutant on rub-inoculated local leaves (Fig 4A and 4B). To confirm this result, 100 *N*. *benthamiana* plants were rub-inoculated in 10 independent experiments with the U178G/U179G mutant, and in each a 100% local leaf infection rate was achieved but systemic infection was never observed. A total of seven progeny clones were recovered from inoculated leaves over the course of several experiments. Sequencing showed that five of the seven progeny retained both mutations. In the remaining two progeny U179G was retained while U178G reverted to wild type. Next, RT-PCR was performed using PSTVd-specific primers with RNA extracted from the petioles of leaves collected 10 days after rub-inoculation. PCR products were readily observed for wild type PSTVd, but no signal was detected in petiole RNA from plants inoculated with U178G/U179G, indicating that the mutant failed to traffic out of local leaves (Fig 4C).

**Fig 5:**
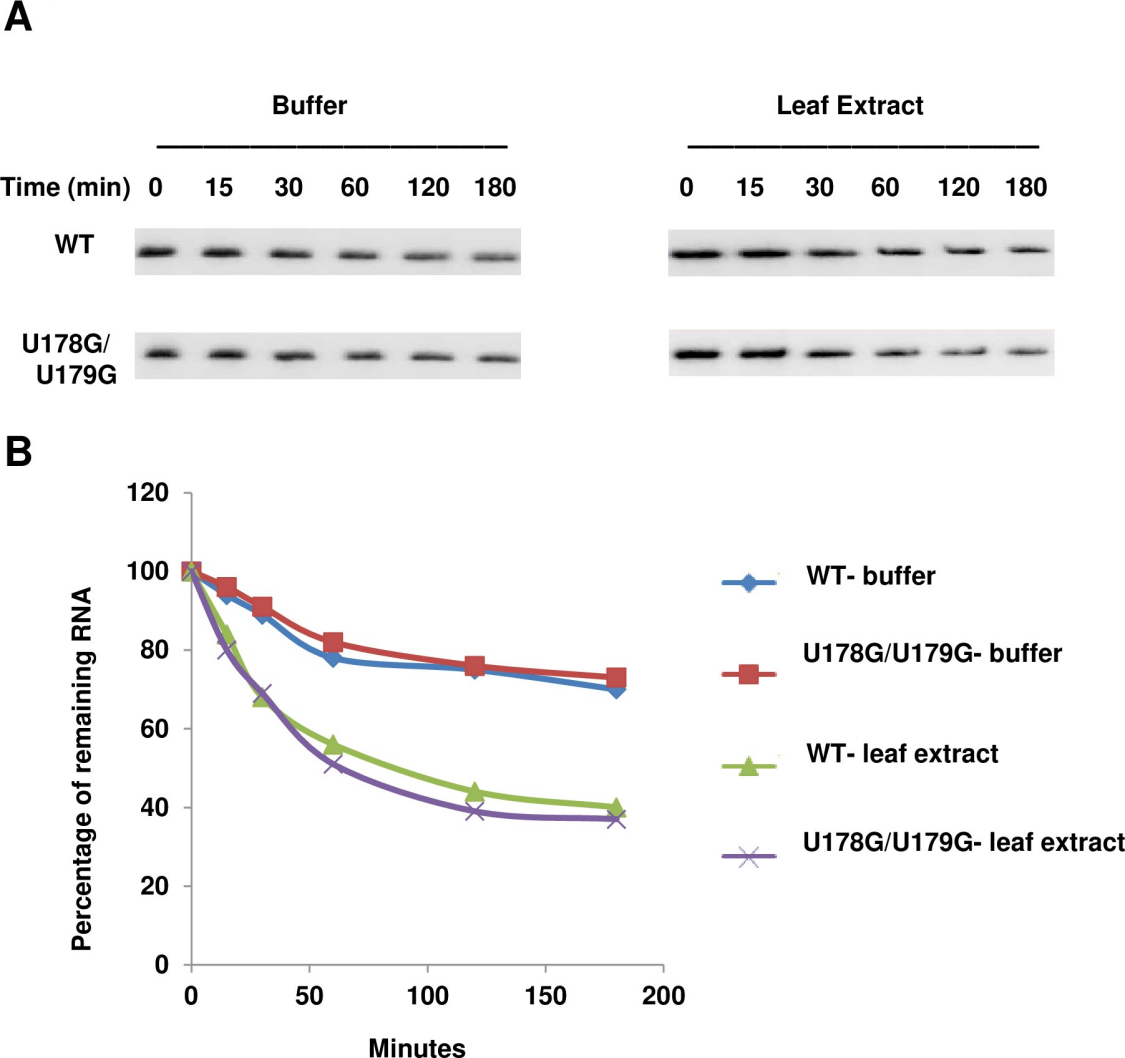
Stability of U178G/U179G is similar to wild type PSTVd. (A) RNA blot of *in vitro* degradation assays performed at 28°C in buffer (20 mM Tris-HCl pH 7.5, 150 mM NaCl, 10 mM phenylmethylsulfonyl fluoride), or uninfected *N*. *benthamiana* leaf extract prepared with the same buffer. (B) Percentage of remaining PSTVd wild type (WT) and U178G/U179G RNA over time was determined using Quantity One software. Data are representative of three independent experiments.

Reduced RNA stability can result in rapid degradation, leading to failure of systemic infection. To assess stability, PSTVd wild type and U178G/U179G RNAs prepared *in vitro* were incubated over time in buffer or in extracts prepared from healthy *N*. *benthamiana* leaves. The amount of RNA remaining at each time point was determined by blot analysis. As expected, degradation occurred more rapidly in leaf extracts than in buffer, but decay rates were similar for both RNAs (Fig 5). While this artificial assay does not mimic the complex cellular milieu in which PSTVd replicates, it nevertheless shows that the mutant RNA is reasonably stable. Thus, it seems unlikely that reduced stability is responsible for the failure of U178G/U179G to spread to upper leaves.

**Fig 6:**
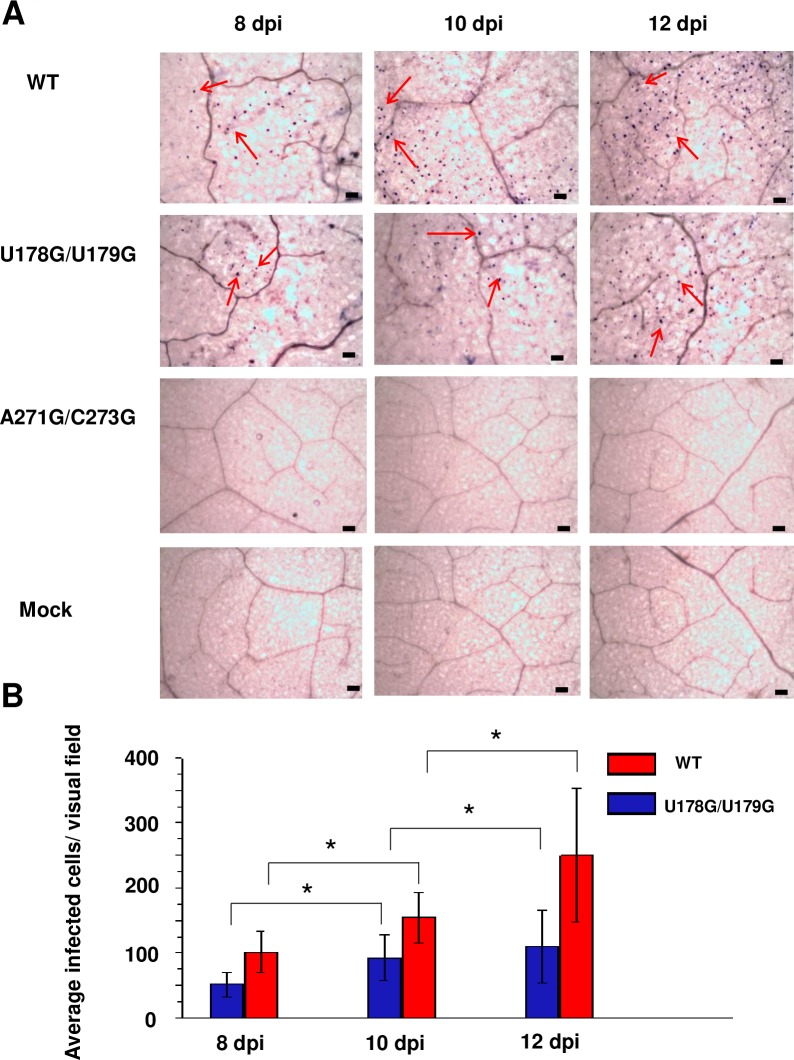
U178G/U179G replicates and spreads in rub-inoculated leaves. (A) Whole-mount *in situ* hybridization was used to monitor infection in leaves rub-inoculated with wild type PSTVd (WT), U178G/U179G, and replication defective A271G/C273G (negative control) at 8, 10, and 12 days post-inoculation (dpi). Mock inoculation was another negative control. Purple dots (red arrows) are viroid hybridization signals in nuclei. Bars = 100 μm. Images for PSTVd WT and U178G/U179G are representative of more than 200 visual fields. (B) Mean numbers of infected cells per visual field after U178G/U179G (blue) and WT (red) inoculation. Asterisks indicate significant differences (p < 0.05) as determined by Student's *t* test. Bars indicate standard error of the mean.

Spread of viruses and viroids in plant hosts occurs in two stages: local cell-to-cell spread via plasmodesmata that interconnect adjacent cells, followed by systemic long-distance transport in vascular tissue, typically the phloem. Thus, an inability to spread cell-to-cell might block systemic trafficking. To address this issue, whole mount *in situ* hybridization using antisense digoxigenin (DIG)-labeled PSTVd riboprobes was carried out with tissue harvested 8, 10, and 12 days after rub-inoculation. Here, whole mount indicates that upper surfaces of small leaf pieces, rather than thin sections, were examined. While this method of observation does not allow unambiguous identification of cell types, most nuclei containing PSTVd signals likely belong to epidermal cells (see below). More than 200 visual fields (~1 mm x 1 mm), randomly selected from inoculated leaves of 10 plants, were examined for each time point. As shown in Fig 6A, positive PSTVd hybridization signals were evident in the nuclei of cells infected with wild type PSTVd or U178G/U179G. No signal was observed in samples from mock inoculated leaves (negative control) or leaves inoculated with an A271G/C273G mutant that is unable to replicate (negative control for residual inoculum). Importantly, a significant increase in the number of infected cells per visual field was observed for wild type PSTVd and U178G/U179G with increased time after inoculation, indicating successful cell-to-cell spread (Fig 6A and 6B). The number of cells infected by U178G/U179G approached 50% of the number infected by wild type PSTVd at each time point (Fig 6B), which is more than sufficient to support systemic trafficking to upper non-inoculated leaves. Previous studies have shown that PSTVd mutants with infection efficiencies as low as 10% of wild type are able to achieve systemic infection [31].

**Fig 7:**
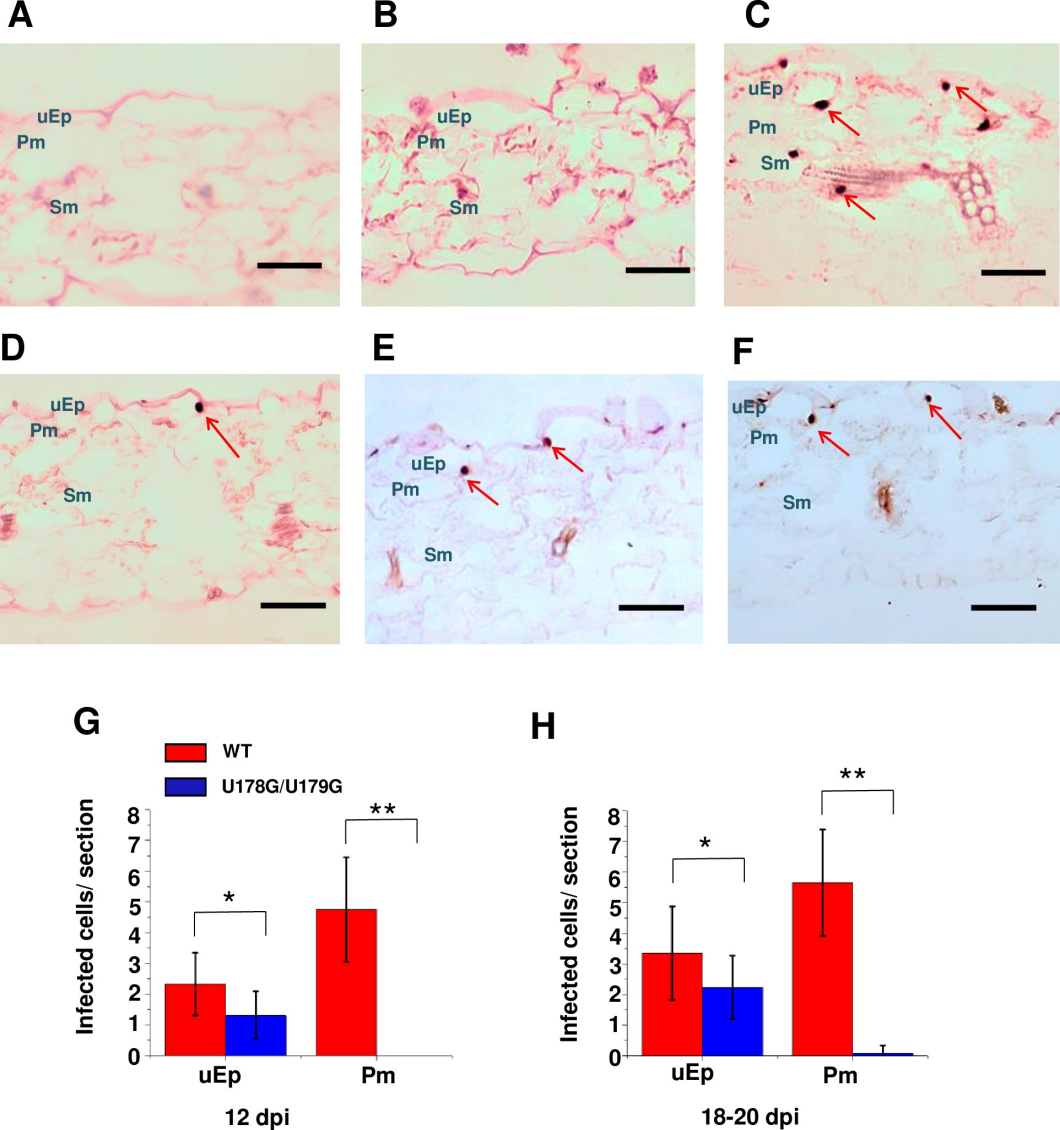
U178G/U179G fails to exit epidermal cells in rub-inoculated leaves. Transverse section (12 μm) *in situ* hybridization of (A) mock inoculated leaves (negative control), (B) leaves inoculated with replication defective A271G/C273G (negative control), (C) wild type PSTVd (positive control), and (D-F) U178G/U179G. Images for PSTVd WT and U178G/U179G are representative of more than 200 sections. Purple dots (red arrows) are viroid hybridization signals in nuclei. uEp, upper epidermis; Pm, palisade mesophyll; Sm, spongy mesophyll; lEp, lower epidermis. Bars = 100 μm. (G and H) Number of infected cells per leaf section (~1 x 0.15 mm) in the upper epidermis (uEp) or adjacent palisade mesophyll (Pm) of plants inoculated with WT PSTVd (red) or U178G/U179G (blue) at 12 dpi (G) and 18–20 dpi (H). Data were compiled from 40 sections obtained from 20 infected plants. Asterisks indicate significant differences (p < 0.05*; p < 0.01**) as determined by Student's *t* test. Bars indicate standard error of the mean.

Spread between different cell types is prerequisite for systemic trafficking. To assess the ability of U178G/U179G to move between different cell types, *in situ* hybridization was performed with transverse sections derived from rub-inoculated leaves. Representative images selected from more than 200 sections obtained from 80 plants are presented in Fig 7. No signal was detected in sections from mock inoculated plants or plants inoculated with replication-defective A271G/C273G (Fig 7A and 7B). As expected, wild type PSTVd was distributed in all cell types (Fig 7C). Surprisingly, positive signals for U178G/U179G were confined to cells of the upper epidermis (Fig 7D–7F), indicating that the mutant is unable to exit inoculated cells of rub-inoculated leaves. The number of upper epidermal cells infected by U178G/U179G increased over time and approached or exceeded 50% the number infected with wild type PSTVd at 12 and 18–20 dpi. However, in U178G/U179G infected plants, essentially no infected cells were detected at these time points in the immediately underlying palisade mesophyll (Fig 7G–7H). Thus, U178G/U179G is unable to exit the upper epidermis following rub-inoculation, but is capable of lateral cell-to-cell spread within this cell layer.

### Needle puncture inoculation enables systemic spread of PSTVd U178G/U179G between different cell types

PSTVd trafficking between cells occurs through plasmodesmata, and plasmodesmal structures linking different cell types may differ, leading to fine regulation of PSTVd trafficking. Therefore, we investigated the outcome of PSTVd U178G/U179G infections that bypass the epidermis-palisade mesophyll boundary. To do this, linear and circular forms of U178G/U179G RNA inoculum were injected into vascular tissues of *N*. *benthamiana* plants by needle puncture of stems and petioles, and leaves above the inoculation sites were collected 28 days later. Subsequent RNA blot analysis indicated that needle puncture delivery of linear forms failed to systemically infect plants (no infections in three experiments, 10 plants each). However, although infection rates were usually less than 30% for both wild type PSTVd and U178G/U179G, needle puncture inoculation of circular forms resulted in successful systemic infection (Table 2). Sequencing of full-length genomic clones obtained from upper leaf extracts showed that 21 of 26 progeny derived from U178G/U179G maintained both original mutations, indicating high genetic stability. Another two progeny also retained U178G/U179G but acquired a one base insertion or a one base deletion at a distal site. In the remaining three progeny U179G was retained while U178G reverted to wild type (Table 2).

**Table 2:** Sequences of progeny from plants inoculated with U178G/U179G by needle puncture.

	WT (U178/U179)	U178G/U179G	U178G/U179G progeny sequences
**Experiment 1**	3/10	3/10	**U178G/U179G (1)**
**Experiment 2**	2/10	2/10	**U178G/U179G (4)** U179G (1)
**Experiment 3**	4/10	1/10	N/A
**Experiment 4**	1/10	2/10	**U178G/U179G (5)** U178G/U179G/190A191 (1)
**Experiment 5**	0/10	1/10	**U178G/U179G (1)**
**Experiment 6**	1/10	0/10	N/A
**Experiment 7**	3/10	2/10	**U178G/U179G (5)** U178G/U179G/△89A (1)
**Experiment 8**	1/10	2/10	**U178G/U179G (5)** U179G (2)

In each experiment, ten plants were inoculated by needle puncture with wild type PSTVd (WT) or U178G/U179G. The number of systemically infected plants, determined by blot assay of RNA collected from upper leaves, is indicated. U178G/U179G progeny clones (26 total) were recovered from systemically infected leaves. The 21 progeny that retained the original double mutation are indicated in bold. Of the remaining five, two retained the double mutation but acquired an additional change (a one base insertion or a one base deletion), and three retained U179G while U178G reverted to wild type. N/A indicates no clones obtained.

**Fig 8:**
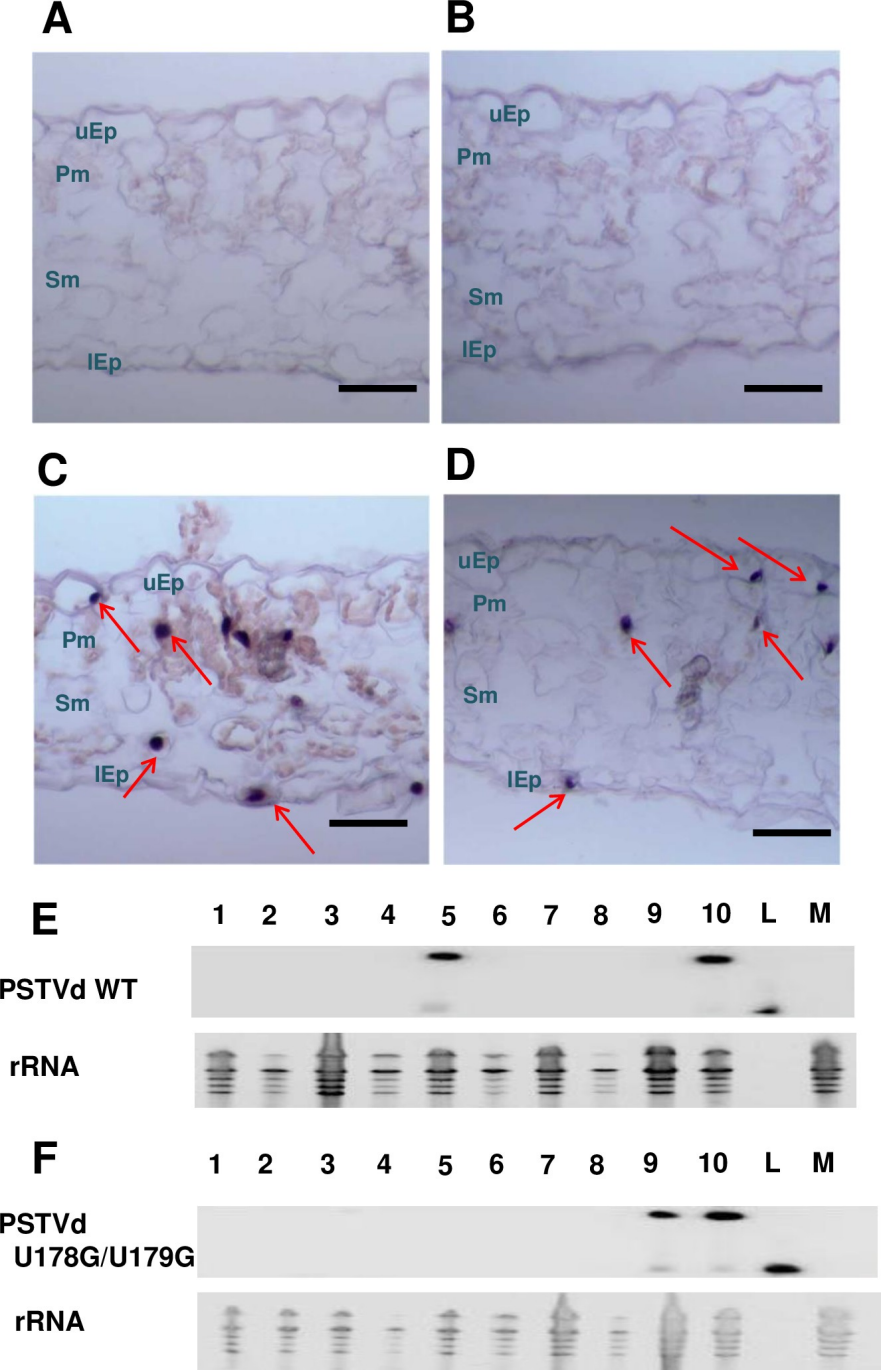
Needle puncture inoculation of stems and petioles allows trafficking of U178G/U179G to all cell types in upper leaves. Transverse section (12 μm) *in situ* hybridization of samples from upper leaves of plants that were: (A) mock inoculated (negative control), or needle puncture inoculated with (B) replication defective A271G/C273G (negative control), (C) wild type PSTVd (positive control), or (D) U178G/U179G. Image in (D) is representative of more than 100 sections. Purple dots (red arrows) are viroid hybridization signals in nuclei. uEp, upper epidermis; Pm, palisade mesophyll; Sm, spongy mesophyll; lEp, lower epidermis. Bars = 100 μm. (E and F) RNA blot analysis. Total RNA was collected from systemically infected leaves of 10 plants inoculated by needle puncture with (E) wild type PSTVd (WT) or (F) U178G/U179G. RNA from mock inoculated plants (M) was a negative control, and linear PSTVd RNA (L) was a hybridization control. Linear PSTVd RNA migrates faster than the circular form that predominates in infected plants. Ribosomal RNA (rRNA) was a loading control. Blots are from Table 2, Experiment 2.

We then performed *in situ* hybridization using transverse sections obtained from systemically infected leaves of needle puncture-inoculated plants. A total of three U178G/U179G-infected plants were selected from Experiments 7 and 8 for this purpose (Table 2), and the image shown in Fig 8 is representative of more than 100 sections. Remarkably, sections obtained from plants infected with wild type PSTVd and U178G/U179G were essentially indistinguishable. Positive signals indicating the presence of U178G/U179G were clearly observed in all cell types, including phloem cells, upper epidermal cells, palisade mesophyll cells, spongy mesophyll cells, and lower epidermal cells.

Taken together, our results indicate that PSTVd U178G/U179G is able to traffic between most cell types, including from palisade mesophyll to upper epidermal cells when delivered into vascular tissue, but is unable to move from infected upper epidermal cells to palisade mesophyll cells following rub-inoculation. Thus, the requirements for transit of PSTVd RNA across the boundary between these cell types are unique and directional, and the U178G/U179G deficiency specifically impacts epidermal exit.

### The U178G/U179G mutant can form a 3D structure resembling the loop 27 model

Both of the cognate single mutants (U178G and U179G) that comprise the atypical double mutant can replicate in inoculated leaves, but U178G does not spread systemically without reversion to wild type. We wondered how a combination of the two mutations results in a PSTVd genome that has nearly wild type function. To address this question, we asked JAR3D to identify a model for the U178G/U179G mutant sequence (5'-UGGUCA-3', loop bases underlined). Unlike wild type Loop 27, which is identical in sequence to HL_4TV0_001 (the histone stem-loop), no identical sequence match was identified for the double mutant. However, U178G/U179G was found to be most compatible with model HL_4OOG_001 (5'-GAGUCC-3'), having a cutoff score of 50.37 (compared to -32.98 with the wild type loop 27 model). HL_4OOG_001 is an RNA-protein co-structure of yeast RNase III with the product of dsRNA processing [48]. The only other mutants that attained a significant positive score with the HL_4OOG_001 model were U178G (7.23) and U179G (38.81).

**Fig 9:**
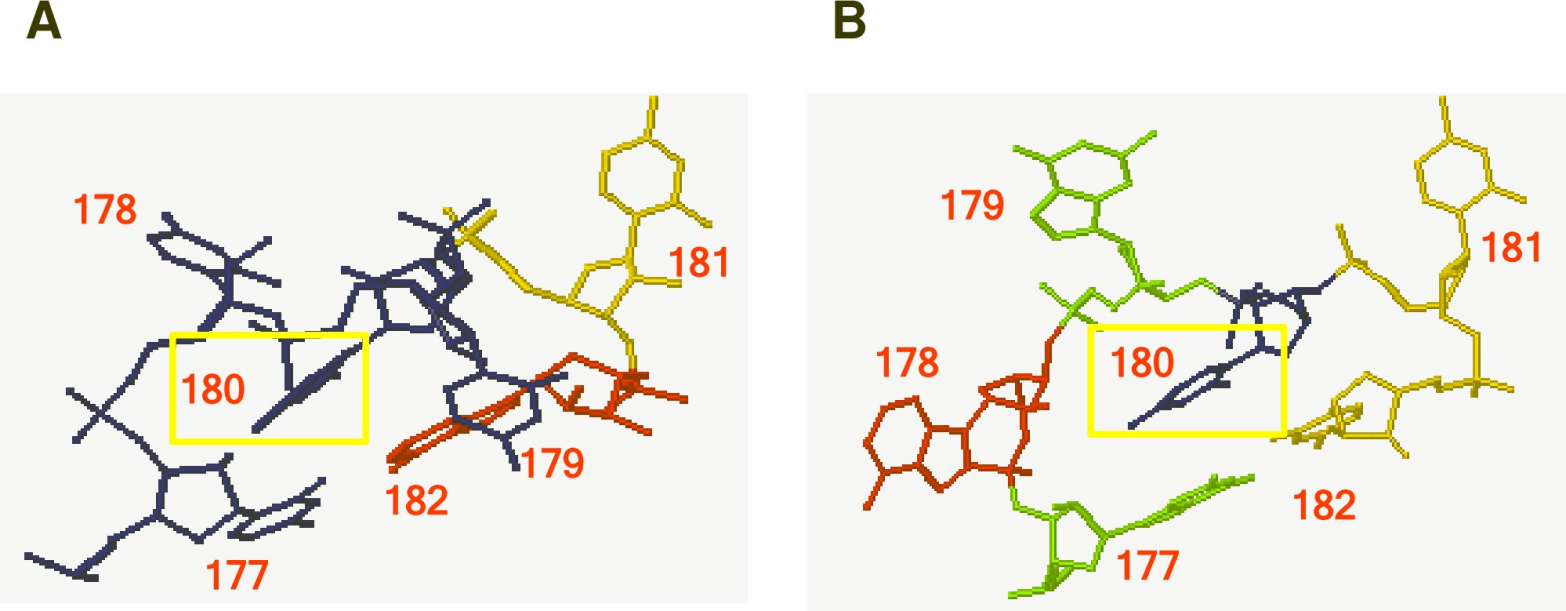
Comparison of loop 27 structural models for wild type PSTVd and the U178G/U179G mutant. (A) Structural model of wild type loop 27 (5'-UUUUCA-3', positions 177–182, loop bases underlined), shown from a different perspective than in Fig 2A. U179 and C181 bulge out of the structure, while U180 (yellow box) stacks on the closing WC base pair between U177 and A182. (B) The predicted structure of the U178G/U179G mutant (5'-UGGUCA-3') is compatible with structural model (HL_4OOG_001; 5'-GAGUCC-3'). The HL_4OOG_001 model structure shown has bulges at positions corresponding to 178, 179, and 181. U180 (yellow box) stacks on the closing WC base pair between G177 and C182.

The U178G/U179G mutant and the HL_4OOG_001 model share three identical bases in the loop (GUC at positions corresponding to 179–181), a purine at position 178 (G vs. A), and a closing WC pair between positions 177 and 182 (U-A vs. G-C). Comparison of the 3D wild type loop 27 model (5'-UUUUCA-3') (Fig 9A) with HL_4OOG_001 (Fig 9B) shows that the former has two bases that bulge out of the structure (U179 and C181), while the latter has three (A178, G179, and C181). However, in both the U at corresponding position 180 stacks on the closing WC base pair, and this residue is important for histone stem-loop/SLBP interaction. Therefore, U178G/U179G may form an alternative structure that shares critical characteristics with wild type loop 27, enabling the mutant to accomplish all functions except epidermal exit. Consistent with this idea, SHAPE probing of the U178G/U179G mutant yielded results similar to wild type loop 27 (S6 Fig).

## Discussion

Extensive X-ray crystallography and nuclear magnetic resonance (NMR) studies have provided overwhelming evidence that nucleotides within the "loops" of correctly predicted RNA secondary structures often form distinct 3D motifs with characteristic non-WC base pairing, base stacking, and backbone interactions. Such motifs serve as platforms for RNA-RNA, RNA-protein, and RNA-small ligand interactions [42, 49, 50]. In this report, we present evidence that PSTVd loop 27 forms a structure similar to the well-characterized histone mRNA 3' UTR stem-loop motif, but performs unique roles in the viroid infection cycle by mediating its replication and spread from epidermal to palisade mesophyll cells.

As predicted by the JAR3D program and verified by DMS and SHAPE probing, the loop 27 model structure (nucleotides 177–182, 5'-UUUUCA-3') consists of a hairpin closed by a WC base pair. The loop contains two bulged bases (U179 and C181) and two bases folded in the structure (U178 and U180), with U180 stacked on the closing U177-A182 pair. Functional mutagenesis based on cutoff scores obtained using JAR3D yielded results consistent with the structural model. Three of the four mutants predicted to disrupt the structure failed to replicate in inoculated leaves. The atypical U178G/U179G mutant was subjected to extensive investigation and is discussed below. Conversely, nine of eleven mutants predicted to be compatible with the model structure were able to replicate. The two mutants that did not replicate affected the closing WC pair (U177C) or the loop base predicted to stack on the closing pair (U180C). Two of the nine replication-competent mutants, double mutants U177A/A182U and U177C/A182G, exchanged the closing U-A pair for A-U or C-G. Interestingly, two additional replication-competent single mutants (U177A and A182G) also perturbed the closing WC pair, and possibly in these cases non-canonical A-A or U-G pairs serve to close the loop. Consistent with this idea, SHAPE probing of U177A and A182 mutant RNAs gave results similar to wild type loop 27 (S6 Fig). (Primary SHAPE data for wild type loop 27 and mutants is shown in S2 Fig.). In any event, with respect to replication in inoculated leaves (and leaving U178G/U179G aside), mutational analysis corroborated JAR3D predictions in 12 of 14 cases and showed that loop 27 structure is necessary but not sufficient to form a replication-competent PSTVd genome. However, JAR3D cutoff scores are not necessarily predictive of function. As cases in point, we note that two of the non-replicating mutants, U177C and U180C, had higher positive cutoff scores than several replication-competent mutants. Thus, although compatible structure is an important factor, sequence must also be considered.

In animal cells SLBP binds the histone stem-loop, and bases important for binding have been experimentally determined (Fig 3A) [35]. Using this interaction as a model, and recognizing that both structure and sequence can be critical for function, mutants predicted to disrupt binding between loop 27 and an as yet unidentified *N*. *benthamiana* protein (or proteins) were also analyzed. Remarkably, only the four mutants predicted to maintain both a structure compatible with the loop 27 model and potential binding sites proved to be systemically infectious following rub-inoculation. These mutations affected the bulged loop bases (U179G, U179C, C181G) or substituted the closing U-A pair with an A-U pair (U177A/A182U). All other replication-competent mutants that affected the closing base pair (U177A, U177G, A182G, U177C/A182G) or loop bases within the structure (U178C, U178G, U180C) either failed to traffic systemically or reverted to wild type. Thus, in addition to an important role in PSTVd replication, loop 27 is also essential for systemic spread when the viroid is introduced by mechanical inoculation.

That mutants retaining the loop 27 model structure and potential binding sites are systemically infectious suggests that *N*. *benthamiana* harbors a protein(s) that binds PSTVd loop 27 in a manner that resembles the SLBP/histone stem-loop interaction in at least some respects. However, there are important differences. First, SLBP binding to the histone stem-loop requires additional bases in a conserved, extended stem that are not present in PSTVd (Fig 3A) [35]. Further, an SLBP homologue in natural PSTVd hosts, including tomato, potato, and tobacco, has not been reported. A BLAST search of the SOL genomics network (https://solgenomics.net/) using human, rat, and zebrafish SLBP amino acid sequences as queries also yielded no promising candidates. There are also clear functional differences. SLBP promotes association of U7 snRNP with histone pre-mRNAs, resulting in 3' end cleavage and maturation [36]. Cleavage and ligation of PSTVd progeny genomes from (+)-strand concatemers generated during replication occurs between nucleotides G95 and G96, well removed from loop 27 [51]. In addition, with a few exceptions including Volvox and Chlamydomonas, plant histone mRNAs are polyadenylated and lack a 3' UTR stem-loop [52, 53]. Mutagenesis instead indicates roles for PSTVd loop 27 in replication and trafficking, and specifically transport across the boundary between epidermal and palisade mesophyll cells. Thus, our studies have revealed novel functions for a conserved RNA structural motif.

Undoubtedly the atypical U178G/U179G mutant proved the most informative of the fifteen examined in this study. Despite a sequence predicted to disrupt both the 3D structure and potential protein binding sites within loop 27, this mutant is replication-competent and able to spread between cells in the upper epidermis following rub-inoculation. And while reduced progeny accumulation levels were observed with U178G/U179G (S1 Table), this is likely due in part to its restriction to the epidermal cell layer. In addition, U178G/U179G can spread to all cell types, including upper epidermis, following needle puncture inoculation of stems and petioles, which allows delivery to vascular tissues. Thus, the loop 27 motif is not needed for trafficking between other cell types, including palisade mesophyll to upper epidermis, but is specifically required for epidermal exit. Interestingly, both of the cognate single mutants (U178G and U179G) can replicate in inoculated leaves, but the U178G mutant does not spread systemically without reversion to wild type. Yet combining the two results in a PSTVd genome that has nearly wild type function, possibly because the double mutant can adopt an alternative structure that shares key features with loop 27.

A previous genome-wide mutational analysis of PSTVd loops indicated that loop 27 is involved in replication and trafficking [54]. Here, we report that in addition to a critical role in replication, loop 27 is necessary for directional trafficking of PSTVd from epidermal cells to palisade mesophyll cells. To our knowledge, this is the first report of an RNA 3D structural motif with this specific function. Previous studies have shown that a PSTVd 3D motif (loop 7) mediates vascular entry [30], and that trafficking from palisade mesophyll to spongy mesophyll in N. benthamiana involves 3D motifs in loop 6 and loop 19 [31–33]. Sequences adjacent to loop 8 and within loop 24 have been shown to constitute a bipartite motif responsible for directional trafficking from bundle sheath to mesophyll cells in young tobacco (*N*. *tabacum*) leaves [29]. Based on this body of evidence, we suggest that distinct RNA 3D motifs mediate trafficking between different cell types [55, 56].

Cell-to-cell movement of PSTVd occurs through plasmodesmata [57], structurally complex channels that provide cytoplasmic continuity between adjacent cells. In addition to small molecules, plasmodesmata mediate the transport of macromolecules, including proteins, RNAs, and RNA-protein complexes [1, 3, 58–60]. Importantly, they are dynamic and undergo changes in permeability that permit transport selectivity, a property that facilitates the elaboration of different cell types, tissues, and organs. They also function as gateways to the vascular system, allowing responses to be coordinated throughout the plant body. Understanding the structure and function of plasmodesmata is a major challenge, and PSTVd continues to be an important tool in the quest to unravel their true nature. From the work noted above, it follows that plasmodesmal gates differ between most and perhaps all cell types. Further, it appears that requirements for passage are often directional, allowing for precise regulation of RNA transport and the establishment of distinct cellular boundaries. In this scenario, the ability of an RNA to pass through a particular gate in a given direction likely involves specific contacts between a 3D RNA structure and a transport and/or plasmodesmal protein.

Viroids that replicate in the nucleus utilize DNA-dependent RNA polymerase II (Pol II) for transcription [61–63]. To date, several proteins that are involved in and/or regulate transcription have been shown to bind PSTVd RNA. Pol II interacts with a region containing loops 1 to 5 [64]. Transcription factor IIIA (TFIIIA, also known as TFIIIA-9ZF) and plant-specific splice variant TFIIIA-7ZF have different roles in the PSTVd infection cycle. TFIIIA-7ZF functions as a Pol II co-factor and interacts with a region encompassing loops 3 and 4. TFIIIA-9ZF, whose precise function is unclear, binds a region containing loop 26 [65]. Interestingly, ribosomal protein L5 (RPL5), a TFIIIA splicing regulator that modulates relative levels of TFIIIA-9ZF:7ZF, also binds PSTVd [66–68]. Binding has been localized to a region critical for replication that includes loop 15 (also known as Loop E) [68]. Finally, viroid RNA binding protein 1 (Virp1), which plays an essential but unknown role in replication, has two binding sites [69, 70]. One spans loop 26 and overlaps the TFIIIA-9ZF binding site, while the second lower affinity site encompasses a region containing loop 23 in PSTV-I. A protein that specifically interacts with loop 27 has yet to be identified. Based on results of our functional mutagenesis, however, we speculate that loop 27 could have multiple interaction partners. One could be a nuclear protein involved in genome transcription or alternatively in mediating import of viroid RNA into the nucleus. Another may be a plasmodesmal component or transport protein that functions at the junction between cells of the upper epidermis and palisade mesophyll. Further research will be required to identify these factors.

In conclusion, it has been demonstrated that most of the 27 PSTVd loops are necessary either for replication or systemic spread [54], and maintaining their 3D structures is likely crucial to PSTVd survival. Thus, RNA 3D structure imposes important constraints on viroid sequence evolution [71], limiting sequence space to bases that maintain a critical structure as well as essential contacts with interacting proteins and ligands.

## Materials and methods

### Chemical probing of loop 27 structure

DMS and SHAPE modification with benzoyl cyanide BzCN, as well as data analysis, were carried out essentially as described by Tijerina *et al*. (2007) [72] and Giguère *et al*. (2014) [44], respectively. PSTVd structure was probed using two genomic strands starting from nucleotide 175 or 321, and three replicates were performed with each strand. Templates for *in vitro* transcription of these strands were prepared by performing reverse transcription using SuperScript III Reverse Transcriptase (ThermoFisher Scientific, Waltham, MA) with RNA extracts obtained from infected plants to generate unit-length cDNAs from circular genomic PSTVd RNA. The reverse transcription primers employed were PSTVd-175: 5'-CTGTTTCGGCGGGAATTAC-3' and PSTVd-321: 5'-GGTAGTAGCCGAAGCGACAG-3 '. This was followed by PCR using a reverse primer and a forward primer with an appended T3 RNA polymerase promoter sequence: PSTVd-175F-5'-GGGGACAAGTTTGTACAAAAAAGCAGAATTAACCCTCACTAAAGGTTTTCACCCTTCCTTT-3' (forward) and PSTVd-175R-5'-GGGGACCACTTTGTACAAGAAAGCTGGGTCCCGAGCTCTGTTTCGGCGGGAATTAC-3' (reverse) for strand 175; PSTVd-321F-5'-GGGGACAAGTTTGTACAAAAAAGCAGAATTAACCCTCACTAAAGGCGAGGGTGTTTAGCC-3' (forward) and PSTVd-321R-5'-GGGGACCACTTTGTACAAGAAAGCTGGGTCCCGAGCTCCGAAGCAAGTAAGATAGAGA-3' (reverse) for strand 321. The resulting products served as template for *in vitro* transcription of unit-length PSTVd using the T3 Megascript kit (ThermoFisher Scientific). Primers used in SHAPE probing reactions were PSTVd-175 and PSTVd-321 listed above. Primers were 5'-end labeled with 5-FAM (50-carboxyfluorescein) for reverse transcription after incubation with BzCN or DMSO (negative control). Primers were 5'-end labeled with HEX (hexachloro-fluorescein) for reverse transcription used to generate sequencing ladders with ddCTP (1.5 mM). QuSHAPE was used to analyze electropherograms [73], and normalized and averaged data were evaluated using RNAstructure (fold tool, soft thermodynamic constraints) [74]. SHAPE for mutants U177A, U178G/U179G, and A182G was performed similarly, except unit-length linear PSTVd transcripts for each mutant (starting point nucleotide 88) were gel purified to serve as template. In collaboration with the EteRNA Project (Lee et al., 2013), SHAPE was also done with NMIA on a PSTVd genome fragment containing Loop 27. Five replicates were performed.

### Construction of PSTVd loop 27 mutants and *in vitro* transcription

Site-directed mutagenesis was performed to generate PSTVd loop 27 mutants using plasmid pRZ6-2 as template and previously described methods [54, 75]. Plasmid pRZ6-2, which contains a cDNA copy of the PSTVd intermediate strain (PSTVd-Int) adjacent to a T7 promoter sequence, was a gift from Dr. Robert Owens. Sequences of primers used to generate loop 27 mutants are listed in S3 Table. Introduced mutations were verified by sequencing mutant plasmid constructs.

Plasmid pRZ6-2-Int and mutant derivatives were linearized with Hind III and employed as templates to generate (+)-PSTVd wild type and mutant *in vitro* transcripts using the T7 Megascript kit (ThermoFisher Scientific). After *in vitro* transcription, DNA template was removed by DNase I treatment, and the MEGAClear kit (ThermoFisher Scientific) was used to purify the transcripts, which served as inoculum for plants and protoplasts. The T7 Maxiscript kit (ThermoFisher Scientific) was used to generate antisense PSTVd riboprobes labeled with [α-^32^P]-UTP or digoxigenin (DIG) using plasmid pInter(-) linearized with SpeI as template [76]. Unincorporated UTP was removed using Sephadex G-25 columns (GE Healthcare Life Sciences, Chicago, IL).

### Plant and protoplast preparation and inoculation

*N*. *benthamiana* plants were grown in the greenhouse of the Biotechnology Support Facility at the Ohio State University. Two weeks after planting, infection was accomplished by rub inoculating the upper surfaces of the first two true leaves that were previously dusted with carborundum powder. PSTVd (+)-strand *in vitro* transcripts (300 ng/plant) in water treated with diethylpyrocarbonate (DEPC) served as inoculum. Wild type PSTVd transcripts were a positive control, and mock inoculation was performed using DEPC-treated water. In some experiments, circularized wild type PSTVd and U178G/U179G transcripts (300 ng/plant) were inoculated by multiple needle puncture injections into *N*. *benthamiana* stems and petioles using a 10 μl syringe (Kloehn #1010, IMI Precision Engineering, Las Vegas, NV). RNA circularization was performed as described by Beaudry and Perrault (1995) [77].

*N*. *benthamiana* protoplasts were prepared and transfected in the presence of polyethylene glycol as described [78]. Protoplasts were transfected with 6 μg (+)-PSTVd transcripts along with 20 μg of a GFP expression plasmid to monitor transfection efficiency.

### RNA extraction and RNA blot hybridization

RNAzol reagent (Sigma-Aldrich, St. Louis, MO) was used to extract total RNA from inoculated leaves and systemic leaves collected at 10 days and 28 days after inoculation, respectively. RNA blot hybridization was performed essentially as described by Zhong *et al*. (2006) [79]. RNA blot signals were quantitated using Quantity One software (BioRad, Hercules, CA), and normalized to wild type controls.

### Sequencing PSTVd progeny

Infected systemic leaves (above the inoculated leaves) were pooled for total RNA extraction using RNAzol reagent (Sigma-Aldrich). In some experiments, RNA was obtained from inoculated leaves. cDNA was prepared by reverse transcription PCR (RT-PCR) essentially as described by Qi and Ding (2002) [76]. The main differences were: i) SuperScript III Reverse Transcriptase (ThermoFisher Scientific) with strand-displacement activity was used to obtain greater than unit-length cDNAs from circular PSTVd progeny genomes; ii) primers used in subsequent PCR reactions allowed amplification of full-length PSTVd cDNA originating from greater than unit-length RT products, but not from linear inoculum. This is because PCR primers spanned the termini of the linear inoculum (S3 Table). PCR reactions were carried out using high fidelity Platinum Taq Polymerase (ThermoFisher Scientific) according to the following protocol: 95°C for 3 minutes; followed by 40 cycles of 95°C for 30 seconds, 55.4°C for 30 seconds, and 72°C for 40 seconds; with a final extension step at 72°C for 10 minutes. PCR products were inserted into a pGEM-T vector (Promega, Madison, WI) for subsequent sequencing.

### RT-PCR detection of PSTVd in petiole RNA

Total RNA extraction from petioles of *N*. *benthamiana* leaves was performed using RNAzol (Sigma-Aldrich). Reverse transcription was performed using Superscript III (ThermoFisher Scientific) with 500 ng of pooled RNA, followed by PCR amplification using Taq polymerase (ThermoFisher Scientific). Actin mRNA was an endogenous control. Sequences of PSTVd PCR primers were: 5'-CGGAACTAAACTCGTGGTTCCT-3' (forward) and 5'-AGGAACCAACTGCGGTTCCA-3' (reverse). Primers used for actin were: 5'-CACCATGGCAGATGGAGAGGATATTCAGCC-3' (forward) and 5'-AGGAACCAACTGCGGTTCCA-3' (reverse). PCR products were subjected to 1.2% agarose gel electrophoresis and visualized by ethidium bromide staining.

### RNA degradation assay

*N*. *benthamiana* leaf extracts were prepared by grinding young leaf tissue (100 mg) in 1 ml buffer (20 mM Tris-HCl, pH 7.5, 150 mM NaCl, 10 mM PMSF), followed by centrifugation to collect the supernatant. RNA degradation assays were performed by mixing *in vitro* PSTVd wild type and U178G/U179G transcripts (10 ng) with the supernatant (90 μL), or buffer only as a control, at 28°C. Samples were taken at 0, 15, 30, 60, 120, and 180 minutes and immediately frozen with liquid nitrogen after collection. RNA blot analysis was performed and signals were quantified using Quantity One software (Bio-Rad, Hercules, CA). Degradation curves were prepared using the quantified data.

### Sample preparation and *in situ* hybridization

Leaf samples were processed as previously described [29, 31, 80], with slight modifications. The main differences were: i) samples were fixed in 3.7% FAA solution (50% ethanol/3.7% formaldehyde/5% acetic acid) overnight at 4°C; ii) samples were hybridized with DIG-labeled PSTVd antisense riboprobes at 55°C overnight. Whole-mount *in situ* hybridization was performed following the protocol described by Traas (2008) [81].

## Supporting information

S1 Fig Conservation of the 5'-UUUUCA-3' stem-loop among viroid species. Presence of the stem-loop in the genomes of 32 viroid species was determined by analysis of sequences and established RNA secondary structures and/or by RNA folding using Mfold. Numbers in red indicate that the stem-loop is present in the majority of variants. NA indicates complete sequence is not available.
(TIF)

S2 Fig Analysis of wild type loop 27 and selected mutants by SHAPE. SHAPE was performed using BzCN as described Materials and Methods. Normalized original data for wild type loop 27 (A) and selected mutants, including U177A (B), U178G/U179G (C), and A182G (D) are shown. For the mutants, unit-length linear PSTVd transcripts (starting point nucleotide 88) were gel purified and incubated with BzCN or DMSO (negative control). Peaks indicate strong stops following reverse transcription using primer PSTVd-321. In parallel, ddCTP was added to reverse transcription reactions containing wild type PSTVd RNA to generate a sequence ladder. Aliquots of completed ddCTP reactions were added to completed BzCN and DMSO reactions, indicated ddCTP (+) and ddCTP (-), respectively, followed by capillary sequencing.
(TIF)

S3 Fig Analysis of loop 27 by SHAPE. PSTVd structure has been probed using three chemicals in four separate studies. The region shown includes terminal loop 27 and the closing U177-A182 pair, the adjacent base-paired stem, and two bases of loop 26. (A) SHAPE reactivity using BzCN from this study (see Fig 2C). (B) SHAPE reactivity using BzCN from Adkar-Purushthama *et al*., 2015 [45]. (C) SHAPE reactivity using NMIA from this study (see Fig 2D). (D) SHAPE reactivity using NMIA from López-Carrasco and Flores, 2017 [47]. (E) SHAPE reactivity using NAI from López-Carrasco and Flores, 2017 [47]. SHAPE reactivity is indicated by color: red = high (>0.85), orange = intermediate (0.40–0.85), black = low (0–0.40). Low reactivity indicates a higher probability of base pairing.
(TIF)

S4 Fig Control showing wild type PSTVd in rub-inoculated (local) leaves. Total RNA was collected from inoculated (local) leaves of 10 plants infected with wild type PSTVd. An RNA blot assay is shown. Mock inoculation (M) was a negative control. Control lanes contained linear inoculum (I) and circular form (C) PSTVd RNAs (1 ng each). Ribosomal RNA (rRNA) stained with ethidium bromide was a loading control. The position of circular progeny genomes in infected plants is noted, as is the linear residual inoculum. Plasmid pRZ6-2 used to prepare inoculum contains a cDNA copy of PSTVd flanked by ribozyme cleavage modules adjacent to a T7 promoter (Hu *et al*., 1997 [75]). Following plasmid linearization and *in vitro* transcription four PSTVd containing products are possible, depending on whether both sites are cleaved, none are cleaved, or cleavage occurs on one side or the other with respect to the PSTVd insertion. Residual inoculum refers to these products. The invariably high infection rate (10/10 plants) of wild type PSTVd in both local (shown here) and systemic leaf assays served as a positive control in all experiments.
(TIF)

S5 Fig Replication of loop 27 mutants in protoplasts. Five mutants that failed to replicate by the inoculated leaf replication assay were transfected to *N*. *benthamiana* protoplasts. Inocula consisted of 6 μg of (+)-PSTVd transcripts together with 20 μg of a GFP-encoding plasmid, which served as an indicator of PEG-mediated transformation efficiency. Wild type (WT) PSTVd was a positive control, and GFP plasmid alone was a negative control. (A) Protoplasts were photographed ~18 hours post-transfection in a fluorescence microscope using a filter to block red chlorophyll autofluorescence and image only GFP-expressing cells (top panel). Green, blue, and red channels were used to visualize all cells (bottom panel). Transfection efficiencies were similar in all cases (~30%). (B) PSTVd RNAs in transfected protoplasts were detected by RNA blot analysis. rRNA, visualized by ethidium bromide staining, was a loading control. All mutants appeared to replicate by this assay. However, progeny sequencing revealed that all had reverted to wild type and acquired new mutations (S2 Table). Images are representative of three independent experiments.
(TIF)

S6 Fig Chemical probing of wild type and mutant loop 27. Shown are BzCN-SHAPE probing results for wild type (WT) loop 27 (A), U177A (B), U178G/U179G (C), and A182G (D). DMS modification results for wild type Loop 27 are shown in (E). The region shown includes terminal loop 27 and the closing U-A pair (U177—A182), the adjacent base-paired stem, and two bases of loop 26. SHAPE and DMS reactivity is indicated by color: red = high (>0.85), orange = intermediate (0.40–0.85), black = low (0–0.40). Low reactivity indicates a higher probability of base pairing. In E, gray indicates G and U bases that are not modified by DMS.
(TIF)

S1 Table Relative replication/accumulation levels of Loop 27 mutants in local and systemic leaves. Relative replication/accumulation levels of PSTVd Loop 27 mutants in individual plants (10 plants inoculated for each experiment) were determined by RNA blot signal density. Signals were normalized relative to averaged wild type PSTVd signals from 10 plants, which served as a control in each experiment. PSTVd levels in inoculated leaves (L, top group) and systemically infected leaves (S, bottom group) are shown. For systemically infected leaves, only genetically stable mutants (i.e. mutations were retained without introduction of new mutations) are included. Considerable plant-to-plant variation is common, even with wild type PSTVd. However, averaged values reveal clear trends, suggesting three general groups: progeny accumulate to wild type or near wild type levels (0.86–1.42), moderately reduced levels (0.60–0.68), or substantially reduced levels (0.26–0.36). The atypical U178G/U179G mutant is highlighted in red. Low level accumulation of this mutant is likely due to its restriction to a single cell layer.
(DOCX)

S2 Table Sequences of progeny genomes obtained from protoplasts transfected with PSTVd loop 27 mutants. In two of three experiments performed, progeny clones were recovered from transfected protoplasts and full-length sequences were obtained. In all cases, introduced mutations had reverted to wild type, and all genomes had acquired new mutations, as indicated.
(DOCX)

S3 Table Primers used to generate PSTVd loop 27 mutants. (DOCX)
