# Correction: A three-dimensional RNA motif mediates directional trafficking of *Potato spindle tuber viroid* from epidermal to palisade mesophyll cells in *Nicotiana benthamiana*

**DOI:** 10.1371/journal.ppat.1010421

**Published:** 2022-03-22

**Authors:** Jian Wu, Neocles B. Leontis, Craig L. Zirbel, David M. Bisaro, Biao Ding

There are errors in some of the negative control image panels in Figs 6 and 7 of this article [1]. Specifically,

- The panel for non-replicating mutant A271G/C273G at 10 days post inoculation (dpi) in Fig 6A is a duplicate of the Mock inoculation panel at 10 dpi in Fig 6A;
- The A271G/C273G panel at 12 dpi in Fig 6A is an inverted duplicate of the Mock panel at 12 dpi in Fig 6A;
- Fig 7A is a scaled and rotated duplicate of Fig 7B.

A revised version of Fig 6A is provided in which the 10 dpi and 12 dpi A271G/C273G panels and all three Mock panels have been replaced. Replacement panels are from replicate images captured during the original experiments. Quantification of infected cells containing replicating PSTVd reported in Fig 6B was carried out prior to and independent of figure preparation, and included only samples from plants infected with wild type PSTVd (positive control) and mutant U178G/U179G. Negative control samples (Mock and A271G/C273G) were not included in quantification because in all cases the number of infected cells was zero. Therefore, the errors in Fig 6A do not impact the quantitative data in Fig 6.

A revised version of Fig 7 is provided in which panels 7A and 7B have been replaced with the correct data from the original experiments. Replacement panels are from replicate images captured during the original experiments. Quantification of infected cells containing replicating PSTVd reported in Fig 7G and 7H was carried out prior to and independent of figure preparation, and included only samples from plants infected with wild type PSTVd (positive control) and mutant U178G/U179G. Negative control samples (Mock and A271G/C273G) were not included in quantification because in all cases the number of infected cells was zero. Therefore, the errors in Fig 7A and 7B do not impact the quantitative data in Fig 7G and 7H.

Underlying data supporting the updated Fig 6A are in S1 File, underlying data for updated Fig 7A–7F are in S2 File, quantitative data tables for Figs 6B, 7G and 7H are in S3 File, underlying data supporting Fig 4C are in S4 File and Figs 8E and 8F are in S5 File.

The authors apologize for the errors in the published article.

Underlying data supporting other results in the published article are available from the corresponding author.

**Fig 6:**
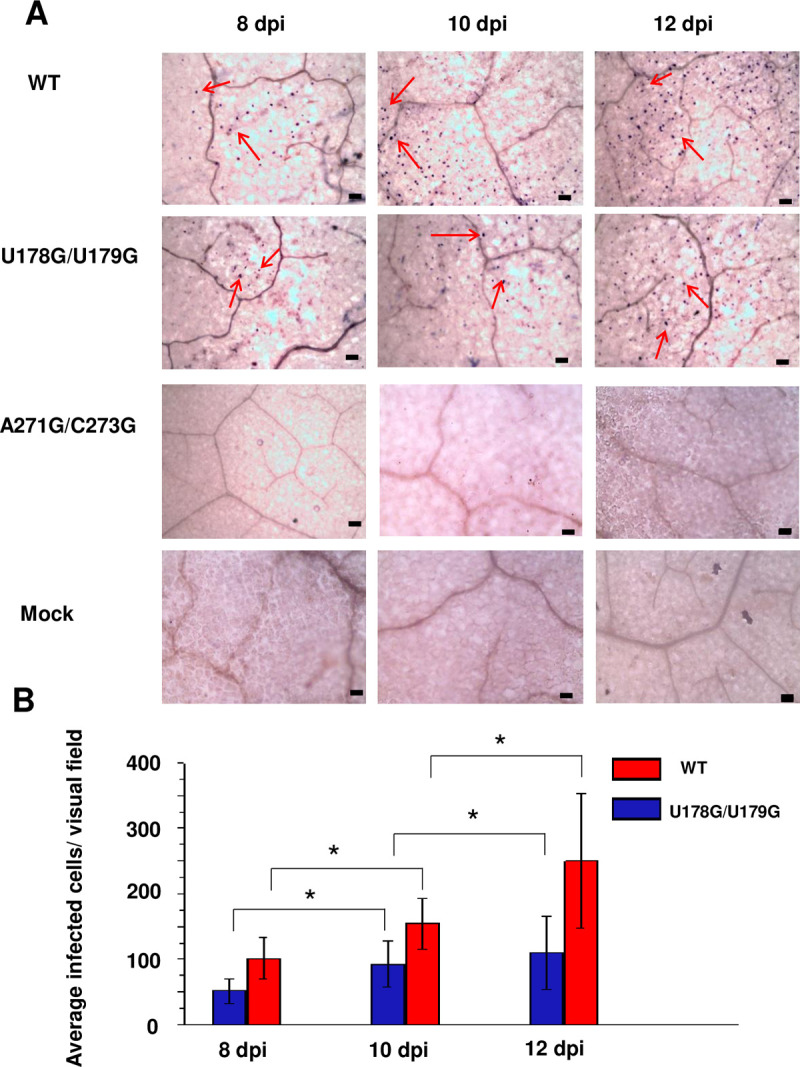
U178G/U179G replicates and spreads in rub-inoculated leaves. (A) Whole-mount *in situ* hybridization was used to monitor infection in leaves rub-inoculated with wild type PSTVd (WT), U178G/U179G, and replication defective A271G/C273G (negative control) at 8, 10, and 12 days post-inoculation (dpi). Mock inoculation was another negative control. Purple dots (red arrows) are viroid hybridization signals in nuclei. Bars = 100 μm. Images for PSTVd WT and U178G/U179G are representative of more than 200 visual fields. (B) Mean numbers of infected cells per visual field after U178G/U179G (blue) and WT (red) inoculation. Asterisks indicate significant differences (p < 0.05) as determined by Student’s *t* test. Bars indicate standard error of the mean.

**Fig 7:**
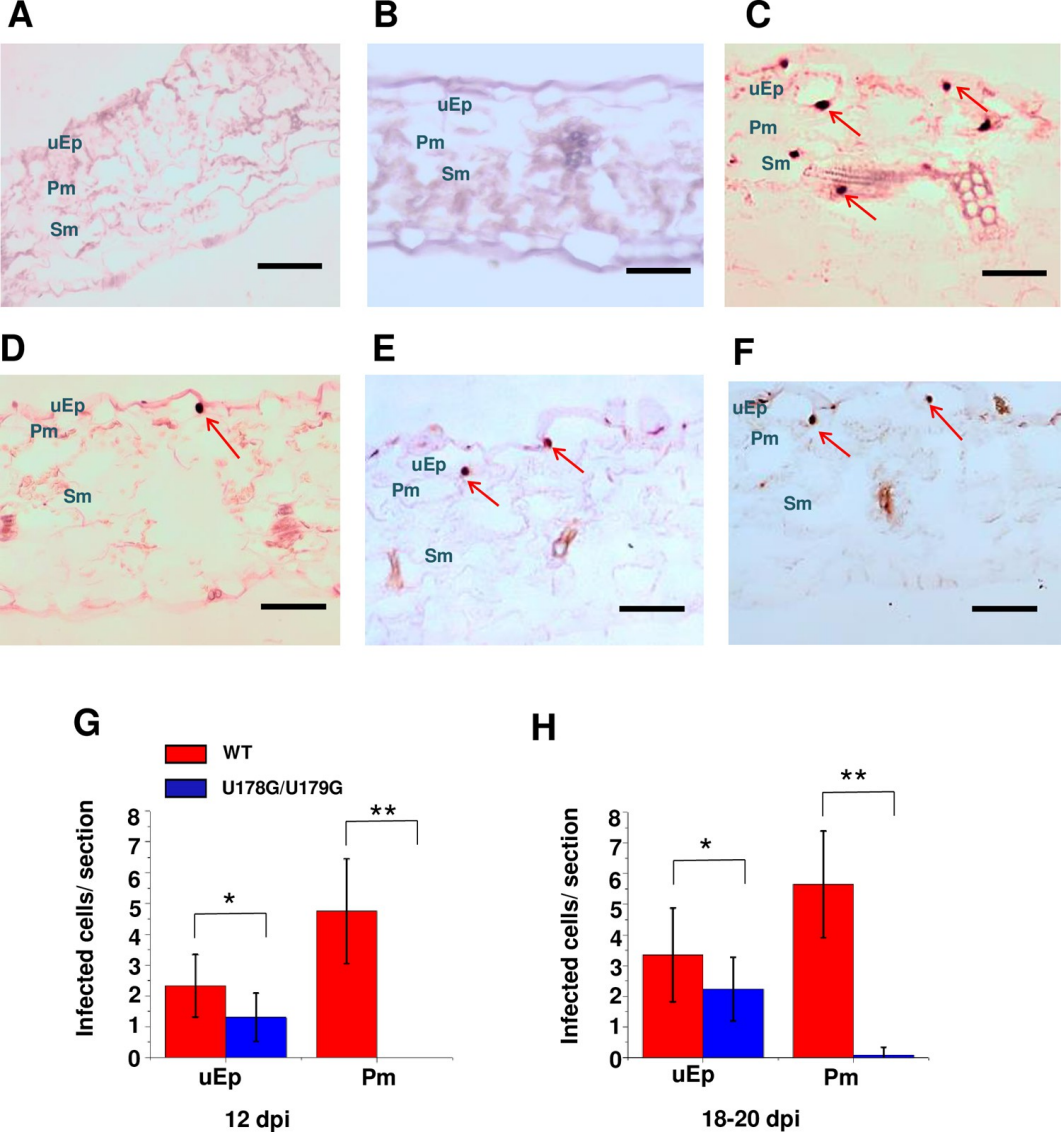
U178G/U179G fails to exit epidermal cells in rub-inoculated leaves. Transverse section (12 μm) *in situ* hybridization of (A) mock inoculated leaves (negative control), (B) leaves inoculated with replication defective A271G/C273G (negative control), (C) wild type PSTVd (positive control), and (D-F) U178G/U179G. Images for PSTVd WT and U178G/U179G are representative of more than 200 sections. Purple dots (red arrows) are viroid hybridization signals in nuclei. uEp, upper epidermis; Pm, palisade mesophyll; Sm, spongy mesophyll; lEp, lower epidermis. Bars = 100 μm. (G and H) Number of infected cells per leaf section (-1 x 0.15 mm) in the upper epidermis (uEp) or adjacent palisade mesophyll (Pm) of plants inoculated with WT PSTVd (red) or U178G/U179G (blue) at 12 dpi (G) and 18–20 dpi (H). Data were compiled from 40 sections obtained from 20 infected plants. Asterisks indicate significant differences (p < 0.05*; p < 0.01**) as determined by Student’s *t* test. Bars indicate standard error of the mean.

## Supporting information

S1 File Underlying data (raw images) for Fig 6. (ZIP)

S2 File Underlying data (raw images) for Fig 7. (ZIP)

S3 File Underlying data (quantitative) for Figs 6 and 7. (ZIP)

S4 File Underlying data (raw images) for Fig 4. (ZIP)

S5 File Underlying data (raw images) for Fig 8. (ZIP)
