# Supplementary figures and images for: A three-dimensional RNA motif mediates directional trafficking of Potato spindle tuber viroid from epidermal to palisade mesophyll cells in Nicotiana benthamiana
Source: PLoS Pathog. 2019 Oct 23;15(10):e1008147. doi: 10.1371/journal.ppat.1008147 (PMC6827988; doi:10.1371/journal.ppat.1008147)

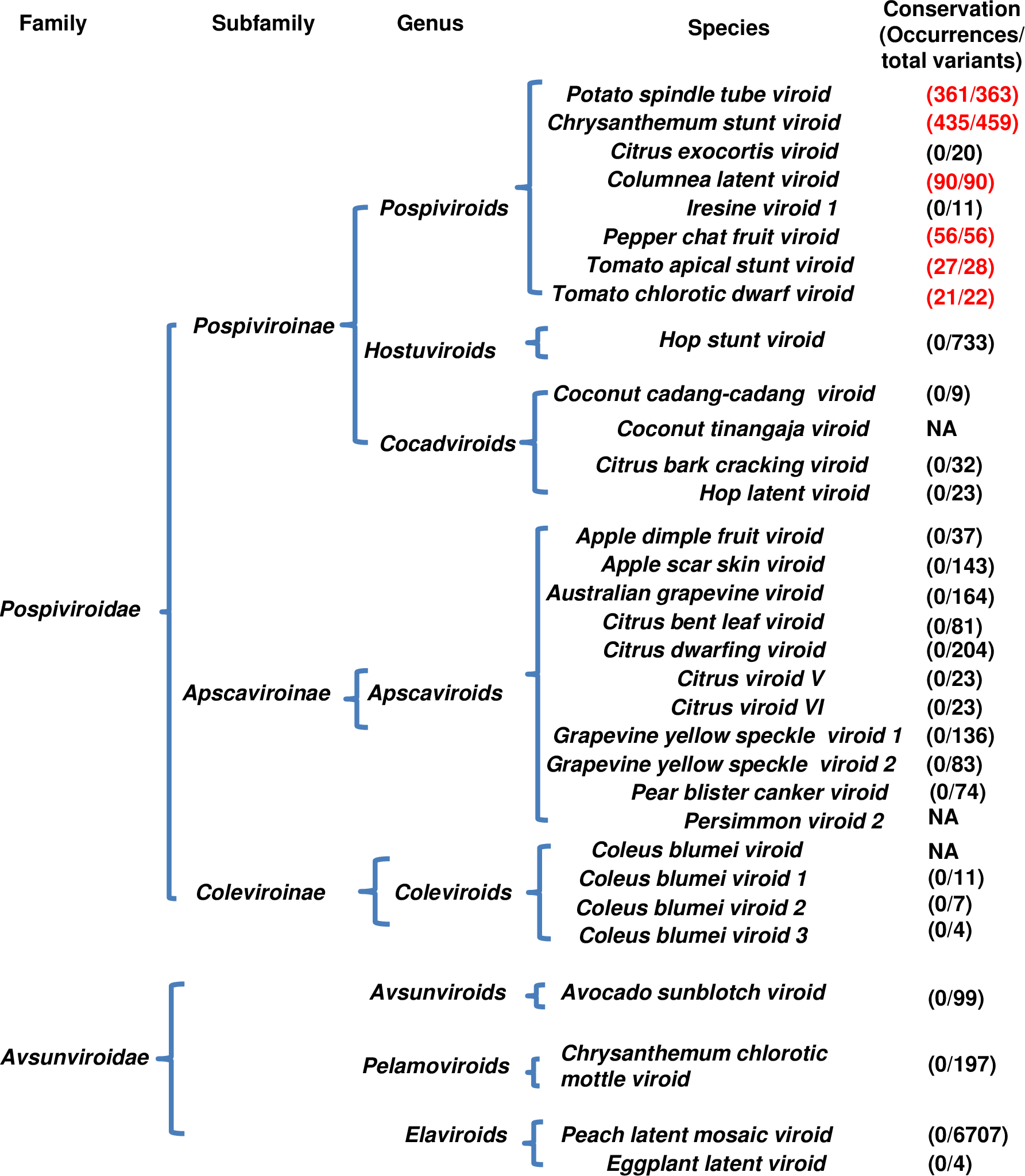

Supplement: S1 Fig — Presence of the stem-loop in the genomes of 32 viroid species was determined by analysis of sequences and established RNA secondary structures and/or by RNA folding using Mfold. Numbers in red indicate that the stem-loop is present in the majority of variants. NA indicates complete sequence is not available. (TIF) [file ppat.1008147.s001.tif]

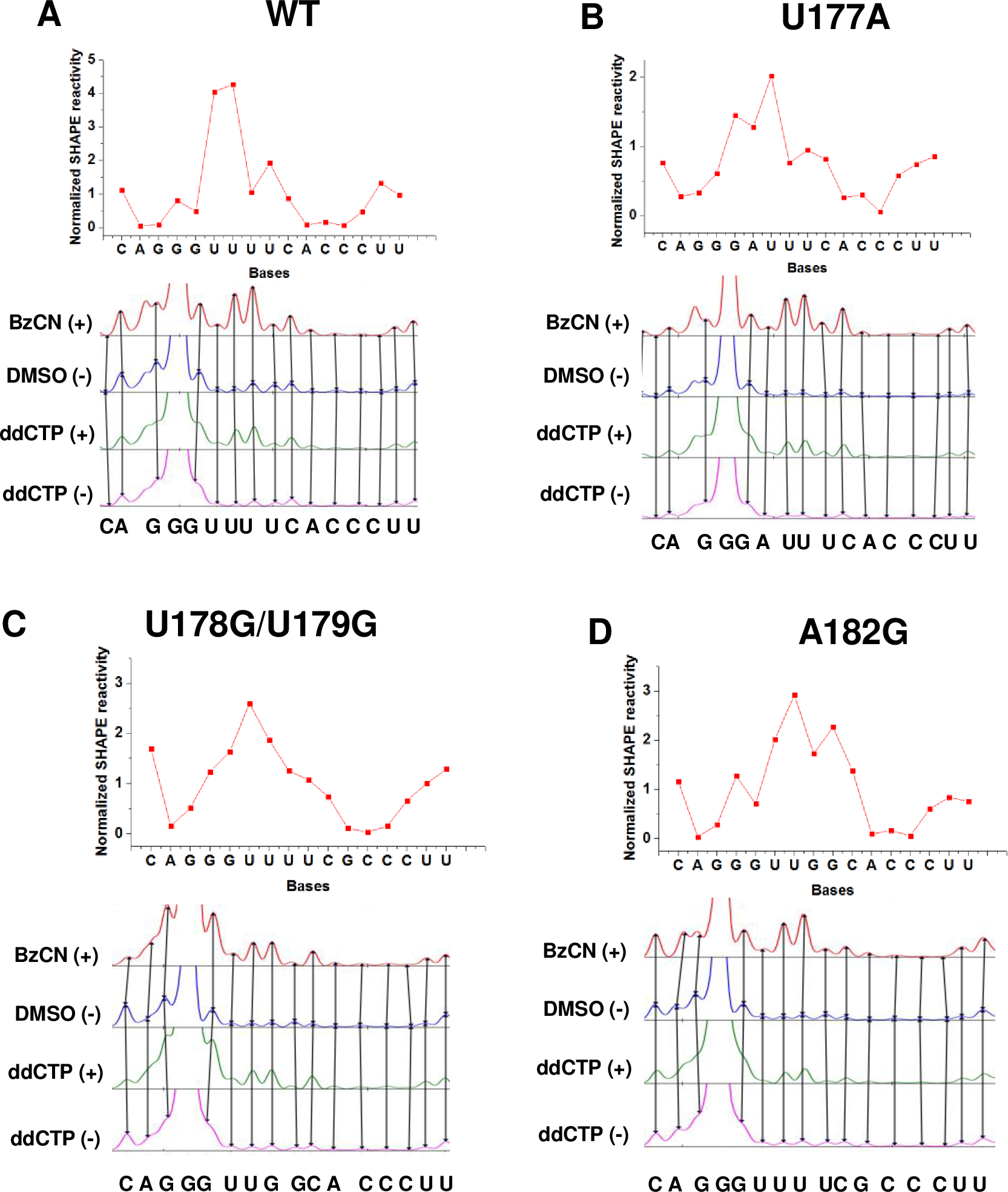

Supplement: S2 Fig — SHAPE was performed using BzCN as described Materials and Methods. Normalized original data for wild type loop 27 (A) and selected mutants, including U177A (B), U178G/U179G (C), and A182G (D) are shown. For the mutants, unit-length linear PSTVd transcripts (starting point nucleotide 88) were gel purified and incubated with BzCN or DMSO (negative control). Peaks indicate strong stops following reverse transcription using primer PSTVd-321. In parallel, ddCTP was added to reverse transcription reactions containing wild type PSTVd RNA to generate a sequence ladder. Aliquots of completed ddCTP reactions were added to completed BzCN and DMSO reactions, indicated ddCTP (+) and ddCTP (-), respectively, followed by capillary sequencing. (TIF) [file ppat.1008147.s002.tif]

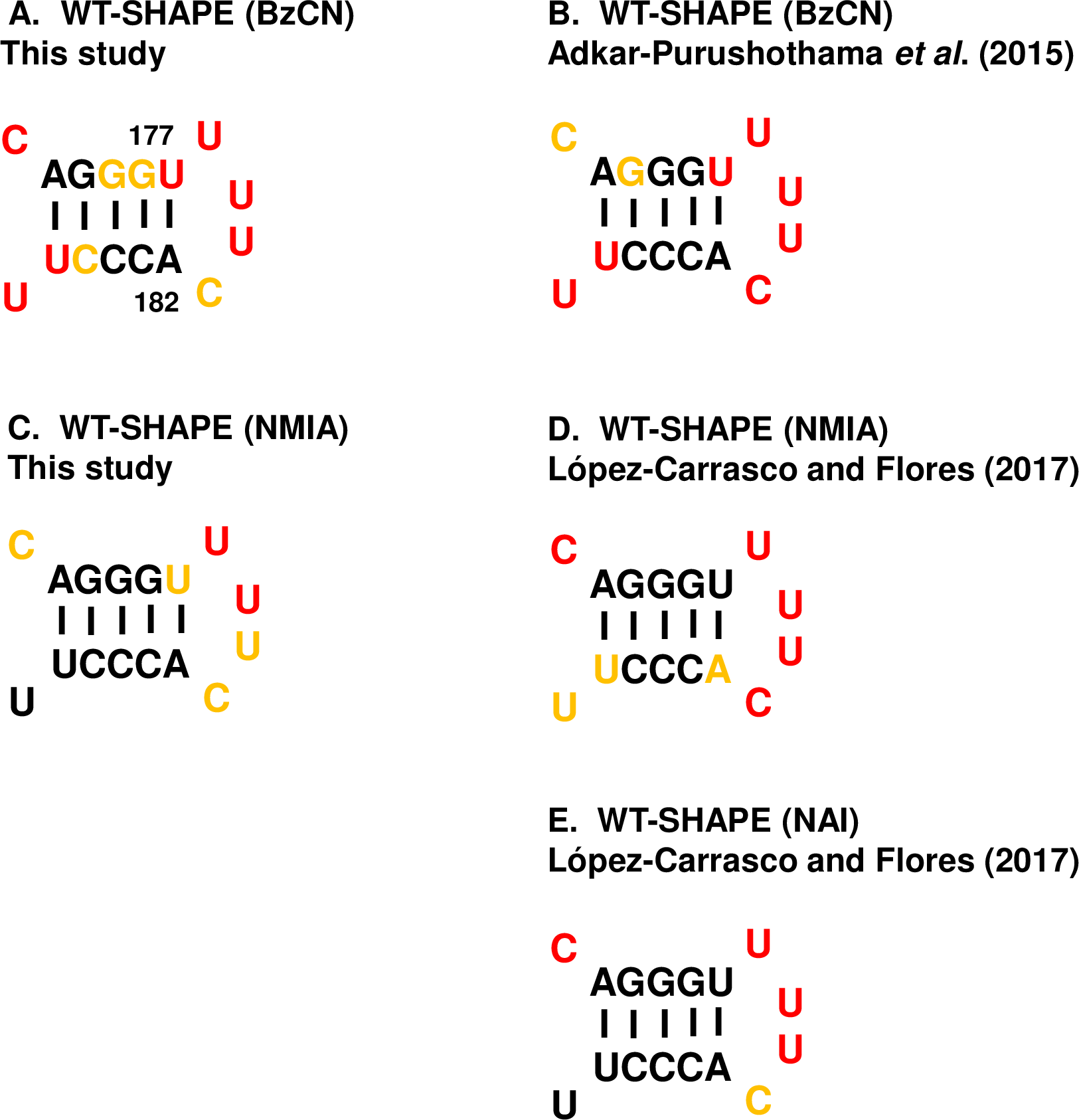

Supplement: S3 Fig — PSTVd structure has been probed using three chemicals in four separate studies. The region shown includes terminal loop 27 and the closing U177-A182 pair, the adjacent base-paired stem, and two bases of loop 26. (A) SHAPE reactivity using BzCN from this study (see Fig 2C). (B) SHAPE reactivity using BzCN from Adkar-Purushthama et al., 2015 [45]. (C) SHAPE reactivity using NMIA from this study (see Fig 2D). (D) SHAPE reactivity using NMIA from López-Carrasco and Flores, 2017 [47]. (E) SHAPE reactivity using NAI from López-Carrasco and Flores, 2017 [47]. SHAPE reactivity is indicated by color: red = high (>0.85), orange = intermediate (0.40–0.85), black = low (0–0.40). Low reactivity indicates a higher probability of base pairing. (TIF) [file ppat.1008147.s003.tif]

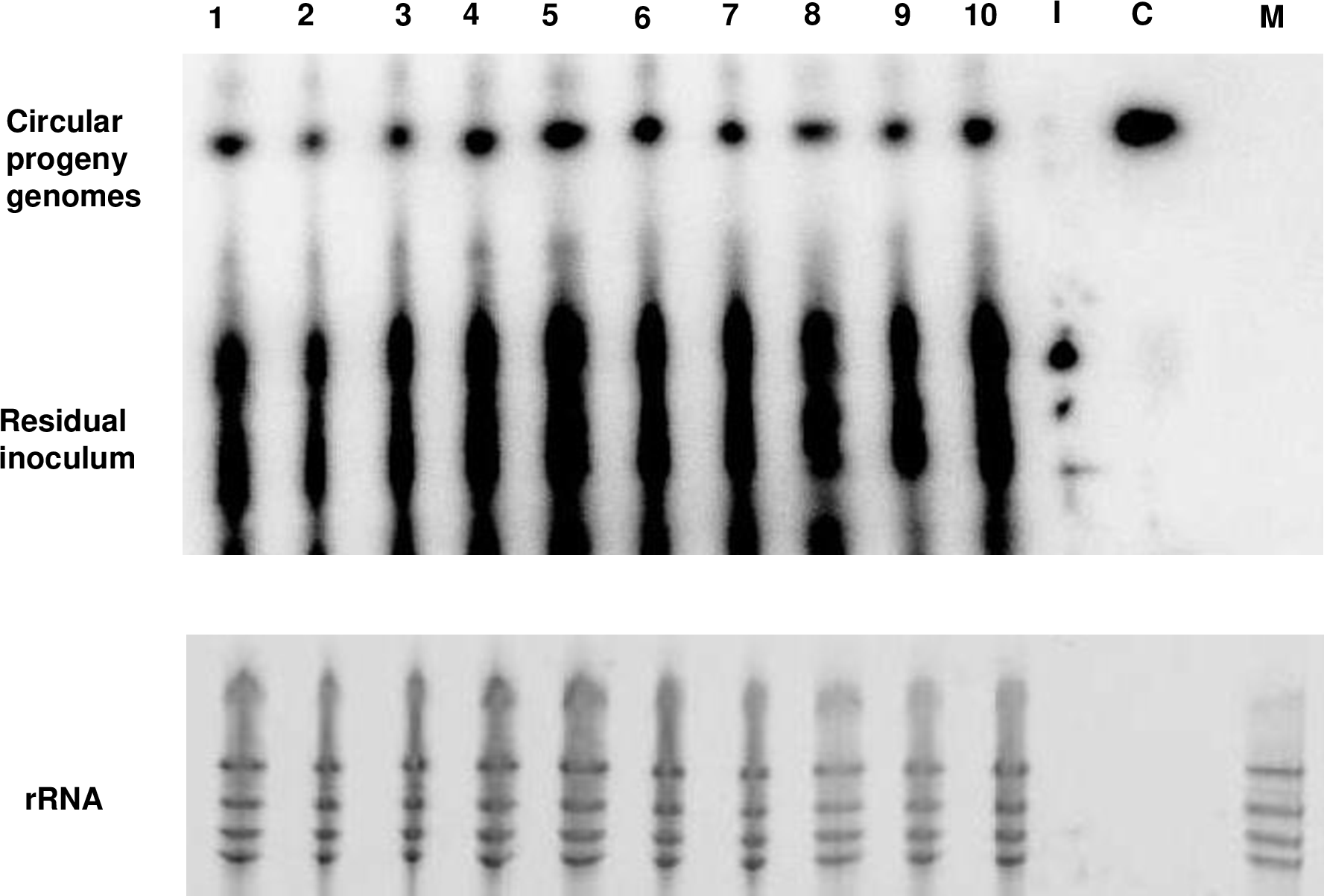

Supplement: S4 Fig — Total RNA was collected from inoculated (local) leaves of 10 plants infected with wild type PSTVd. An RNA blot assay is shown. Mock inoculation (M) was a negative control. Control lanes contained linear inoculum (I) and circular form (C) PSTVd RNAs (1 ng each). Ribosomal RNA (rRNA) stained with ethidium bromide was a loading control. The position of circular progeny genomes in infected plants is noted, as is the linear residual inoculum. Plasmid pRZ6-2 used to prepare inoculum contains a cDNA copy of PSTVd flanked by ribozyme cleavage modules adjacent to a T7 promoter (Hu et al., 1997 [75]). Following plasmid linearization and in vitro transcription four PSTVd containing products are possible, depending on whether both sites are cleaved, none are cleaved, or cleavage occurs on one side or the other with respect to the PSTVd insertion. Residual inoculum refers to these products. The invariably high infection rate (10/10 plants) of wild type PSTVd in both local (shown here) and systemic leaf assays served as a positive control in all experiments. (TIF) [file ppat.1008147.s004.tif]

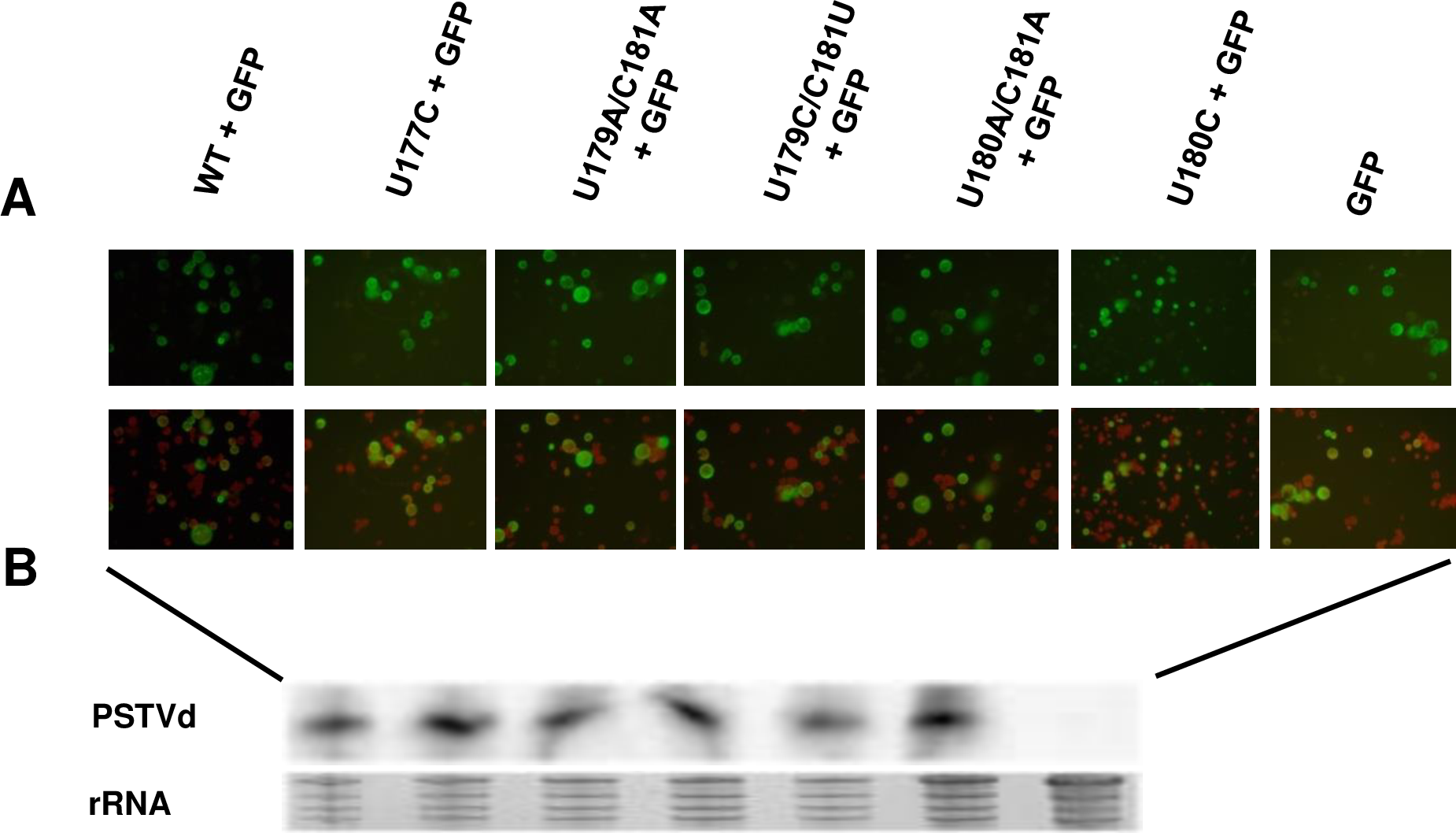

Supplement: S5 Fig — Five mutants that failed to replicate by the inoculated leaf replication assay were transfected to N. benthamiana protoplasts. Inocula consisted of 6 μg of (+)-PSTVd transcripts together with 20 μg of a GFP-encoding plasmid, which served as an indicator of PEG-mediated transformation efficiency. Wild type (WT) PSTVd was a positive control, and GFP plasmid alone was a negative control. (A) Protoplasts were photographed ~18 hours post-transfection in a fluorescence microscope using a filter to block red chlorophyll autofluorescence and image only GFP-expressing cells (top panel). Green, blue, and red channels were used to visualize all cells (bottom panel). Transfection efficiencies were similar in all cases (~30%). (B) PSTVd RNAs in transfected protoplasts were detected by RNA blot analysis. rRNA, visualized by ethidium bromide staining, was a loading control. All mutants appeared to replicate by this assay. However, progeny sequencing revealed that all had reverted to wild type and acquired new mutations (S2 Table). Images are representative of three independent experiments. (TIF) [file ppat.1008147.s005.tif]

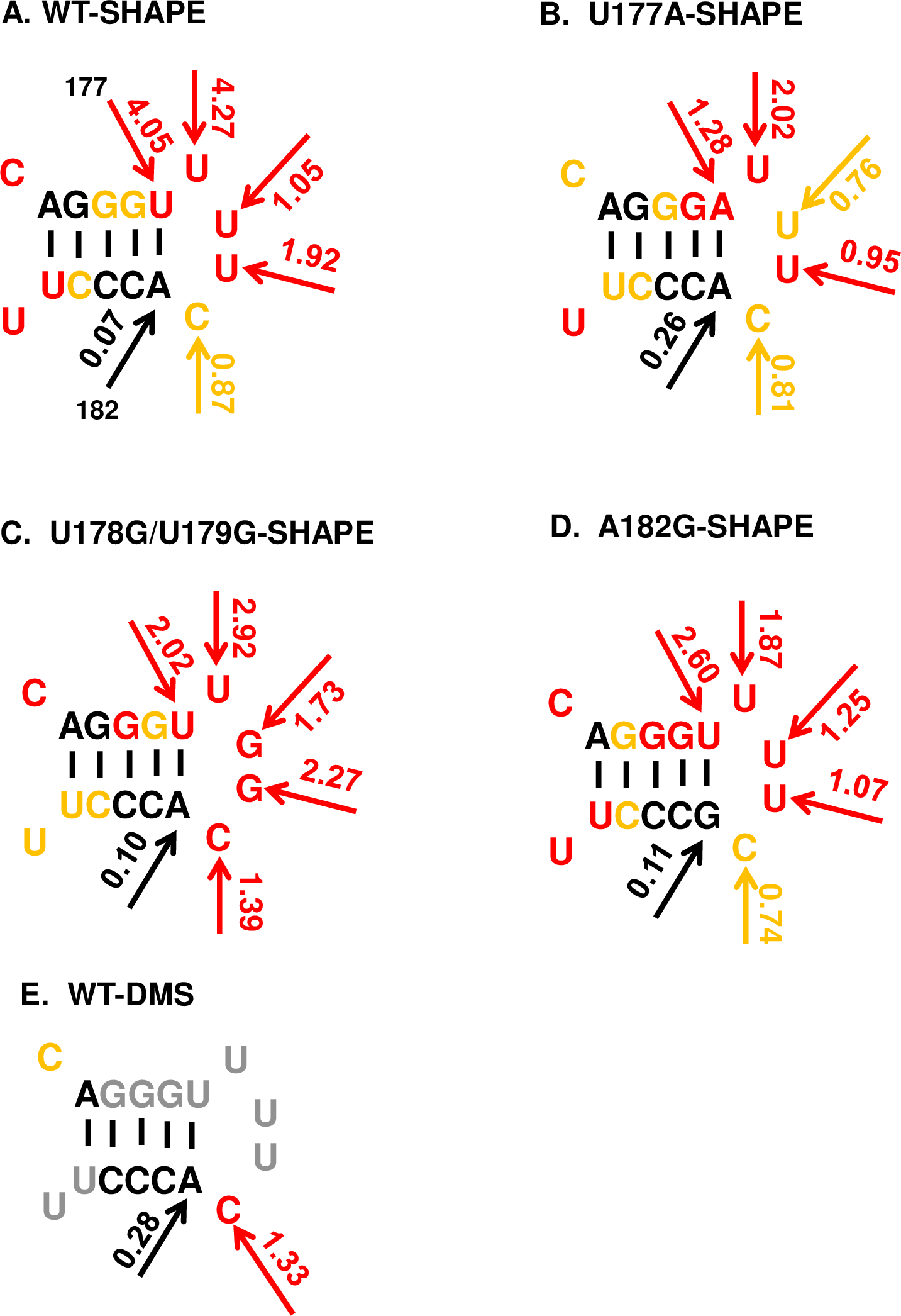

Supplement: S6 Fig — Shown are BzCN-SHAPE probing results for wild type (WT) loop 27 (A), U177A (B), U178G/U179G (C), and A182G (D). DMS modification results for wild type Loop 27 are shown in (E). The region shown includes terminal loop 27 and the closing U-A pair (U177—A182), the adjacent base-paired stem, and two bases of loop 26. SHAPE and DMS reactivity is indicated by color: red = high (>0.85), orange = intermediate (0.40–0.85), black = low (0–0.40). Low reactivity indicates a higher probability of base pairing. In E, gray indicates G and U bases that are not modified by DMS. (TIF) [file ppat.1008147.s006.tif]
