# Supplementary material for: A three-dimensional RNA motif mediates directional trafficking of Potato spindle tuber viroid from epidermal to palisade mesophyll cells in Nicotiana benthamiana
Source: PLoS Pathog. 2019 Oct 23;15(10):e1008147. doi: 10.1371/journal.ppat.1008147 (PMC6827988; doi:10.1371/journal.ppat.1008147)
Supplement: S1 Table — Relative replication/accumulation levels of PSTVd Loop 27 mutants in individual plants (10 plants inoculated for each experiment) were determined by RNA blot signal density. Signals were normalized relative to averaged wild type PSTVd signals from 10 plants, which served as a control in each experiment. PSTVd levels in inoculated leaves (L, top group) and systemically infected leaves (S, bottom group) are shown. For systemically infected leaves, only genetically stable mutants (i.e. mutations were retained without introduction of new mutations) are included. Considerable plant-to-plant variation is common, even with wild type PSTVd. However, averaged values reveal clear trends, suggesting three general groups: progeny accumulate to wild type or near wild type levels (0.86–1.42), moderately reduced levels (0.60–0.68), or substantially reduced levels (0.26–0.36). The atypical U178G/U179G mutant is highlighted in red. Low level accumulation of this mutant is likely due to its restriction to a single cell layer. (DOCX) [file ppat.1008147.s007.docx]

**S1 Table.** Relative replication/accumulation levels of Loop 27 mutants in local and systemic leaves

| **Mutants** | **Infected plants** | | | | | | | | | | |
| --- | --- | --- | --- | --- | --- | --- | --- | --- | --- | --- | --- |
|  | **1** | **2** | **3** | **4** | **5** | **6** | **7** | **8** | **9** | **10** | **Average** |
| U179G-L | 0.46 | 0.55 | 0.49 | 0.62 | 0.56 | 0.61 | 0.73 | 0.26 | 0.74 | 0.95 | 0.60 |
| U179C-L | 1.34 | 1.05 | 1.14 | 1.33 | 1.19 | 0.71 | 1.56 | 0.87 | 1.09 | 1.57 | 1.19 |
| C181G-L | 0.54 | 0.72 | 0.76 | 0.54 | 0.53 | 0.6 | 0.6 | 0.93 | 0.82 | 0.74 | 0.68 |
| U177A/ A182U-L | 1.05 | 1.21 | 1.55 | 1.53 | 1.51 | 1.61 | 1.27 | 1.67 | 1.55 | 1.27 | 1.42 |
| U177A-L | 1.24 | 0.92 | 1.21 | 0.65 | 0.47 | 0.26 | 1.11 | 1.5 | 0.39 | ---- | 0.86 |
| U178C-L | 0.32 | 0.18 | 0.4 | 0.41 | 0.31 | 0.31 | 0.33 | 0.28 | 0.35 | 0.65 | 0.35 |
| U178G-L | 0.36 | 0.22 | 0.42 | 0.49 | 0.31 | 0.29 | 0.33 | 0.3 | 0.27 | 0.63 | 0.36 |
| A182G-L | 0.66 | 0.58 | 0.34 | 0.4 | 0.99 | 0.73 | 0.42 | 0.63 | 1.19 | 0.69 | 0.66 |
| U177C/ A182G-L | 0.83 | 0.81 | 1.15 | 1.26 | 0.97 | 0.8 | 0.74 | ---- | ---- | ---- | 0.94 |
| U178G/ U179G-L | 0.13 | 0.06 | 0.16 | 0.06 | 0.1 | 0.22 | 0.85 | 0.43 | 0.15 | 0.41 | 0.26 |
|  |  |  |  |  |  |  |  |  |  |  |  |
| U179G-S | 0.74 | 0.66 | 0.12 | 1.35 | 1.85 | 1.54 | 0.7 | 0.55 | 0.57 | ---- | 0.90 |
| U179C-S | 2.18 | 0.4 | 1.06 | 0.5 | 0.76 | 0.72 | 0.34 | 1.34 | 0.79 | 0.5 | 0.86 |
| C181G-S | 0.46 | 0.67 | 0.77 | 0.63 | 0.68 | 0.68 | 0.77 | 0.66 | 0.66 | 0.65 | 0.66 |
| U177A/ A182U-S | 1 | 1.12 | 1 | 0.99 | 1.23 | 1.07 | 1.01 | 1.29 | 0.95 | 1.42 | 1.11 |

Relative replication/accumulation levels of PSTVd Loop 27 mutants in individual plants (10 plants inoculated for each experiment) were determined by RNA blot signal density. Signals were normalized relative to averaged wild type PSTVd signals from 10 plants, which served as a control in each experiment. PSTVd levels in inoculated leaves (L, top group) and systemically infected leaves (S, bottom group) are shown. For systemically infected leaves, only genetically stable mutants (i.e. mutations were retained without introduction of new mutations) are included. Considerable plant-to-plant variation is common, even with wild type PSTVd. However, averaged values reveal clear trends, suggesting three general groups: progeny accumulate to wild type or near wild type levels (0.86 – 1.42), moderately reduced levels (0.60 – 0.68), or substantially reduced levels (0.26 - 0.36). The atypical U178G/U179G mutant is highlighted in red. Low level accumulation of this mutant is likely due to its restriction to a single cell layer.
