# Supplementary material for: A three-dimensional RNA motif mediates directional trafficking of Potato spindle tuber viroid from epidermal to palisade mesophyll cells in Nicotiana benthamiana
Source: PLoS Pathog. 2019 Oct 23;15(10):e1008147. doi: 10.1371/journal.ppat.1008147 (PMC6827988; doi:10.1371/journal.ppat.1008147)
Supplement: S2 Table — In two of three experiments performed, progeny clones were recovered from transfected protoplasts and full-length sequences were obtained. In all cases, introduced mutations had reverted to wild type, and all genomes had acquired new mutations, as indicated. (DOCX) [file ppat.1008147.s008.docx]

**S2 Table.** Sequences of progeny genomes obtained from protoplasts transfected with PSTVd loop 27 mutants

|  | **Progeny sequences** | |
| --- | --- | --- |
| **Mutants** | **Experiment 1** | **Experiment 2** |
| U177C | C117A/A121G/U240C/G243U | G138C/A150C |
|  |  | G68A/U157C/C282U |
| U179A/C181A | U191G+17nt deletion | U177A/C259U |
|  |  | C42G/U177A/C259U |
| U179C/C181U | U82C/U83C/A85G | U177A/C258G/U332A |
|  |  | A4G/C41G/C338U |
|  |  | A32C/C259U/C327U/U351C |
| U180A/C181A | G176U/U195C | G246A |
|  |  | A121C/A126G/A150G/G154A |
|  |  | A222U/U352C |
| U180C | C215U/C218G/A274C | A150C/U161C/A344U/C13U |

In two of three experiments performed, progeny clones were recovered from transfected protoplasts and full-length sequences were obtained. In all cases, introduced mutations had reverted to wild type, and all genomes had acquired new mutations, as indicated.
