# Supplementary material for: A three-dimensional RNA motif mediates directional trafficking of Potato spindle tuber viroid from epidermal to palisade mesophyll cells in Nicotiana benthamiana
Source: PLoS Pathog. 2019 Oct 23;15(10):e1008147. doi: 10.1371/journal.ppat.1008147 (PMC6827988; doi:10.1371/journal.ppat.1008147)
Supplement: S3 Table — (DOCX) [file ppat.1008147.s009.docx]

**S3 Table.** Primers used to generate PSTVd loop 27 mutants

|  | **Primer sequences** | |
| --- | --- | --- |
| **Mutants** | **Forward (5'-3')** | **Reverse (5'-3')** |
| U177A | CGAAACAGGGATTTCACCCTTC | GAAGGGTGAAATCCCTGTTTCG |
| U177C | CGAAACAGGGCTTTCACCCTTC | GAAGGGTGAAAGCCCTGTTTCG |
| U178G | CCGAAACAGGGTGTTCACCCTTCCTT | AAGGAAGGGTGAACACCCTGTTTCGG |
| U178C | CGAAACAGGGTCTTCACCCTTCC | GGAAGGGTGAAGACCCTGTTTCG |
| U179G | CCGAAACAGGGTTGTCACCCTTCCTTTC | GAAAGGAAGGGTGACAACCCTGTTTCGG |
| U179C | GAAACAGGGTTCTCACCCTTCC | GGAAGGGTGAGAACCCTGTTTC |
| U180C | CGAAACAGGGTTTCCACCCTTCCTTT | AAAGGAAGGGTGGAAACCCTGTTTCG |
| C181G | AAACAGGGTTTTGACCCTTCCTTT | AAAGGAAGGGTCAAAACCCTGTTT |
| A182G | AACAGGGTTTTCGCCCTTCCTTTC | GAAAGGAAGGGCGAAAACCCTGTT |
| U177A/A182U | ACAGGGATTTCTCCCTTCCTTT | AAAGGAAGGGAGAAATCCCTGT |
| U177C/A182G | ACAGGGCTTTCGCCCTTCCTTT | AAAGGAAGGGCGAAAGCCCTGT |
| U178G/U179G | CGAAACAGGGTGGTCACCCTTCCT | AGGAAGGGTGACCACCCTGTTTCG |
| U179A/C181A | CCGAAACAGGGTTTTCACCCTTCCTT | AAGGAAGGGTGAAAACCCTGTTTCGG |
| U179C/C181U | CCGAAACAGGGTTGTCACCCTTCCTTTC | GAAAGGAAGGGTGACAACCCTGTTTCGG |
| U180A/C181A | CCGAAACAGGGTTTAAACCCTTCCTTTCT | AGAAAGGAAGGGTTTAAACCCTGTTTCGG |
