# Supplementary figures and images for: Correction: A three-dimensional RNA motif mediates directional trafficking of Potato spindle tuber viroid from epidermal to palisade mesophyll cells in Nicotiana benthamiana
Source: PLoS Pathog. 2022 Mar 22;18(3):e1010421. doi: 10.1371/journal.ppat.1010421 (PMC8939776; doi:10.1371/journal.ppat.1010421)

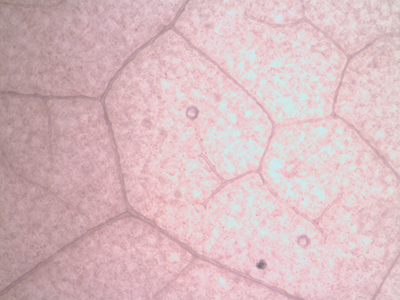

Supplement: S1 File — (ZIP) [file ppat.1010421.s001.zip › Fig 6 Images/A127G-C273G 8dpi.tif]

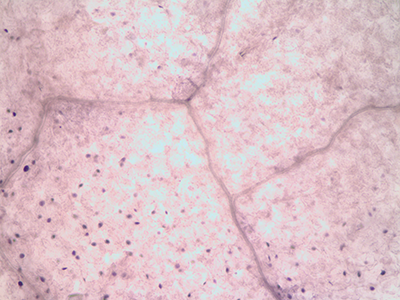

Supplement: S1 File — (ZIP) [file ppat.1010421.s001.zip › Fig 6 Images/WT 10dpi.tif]

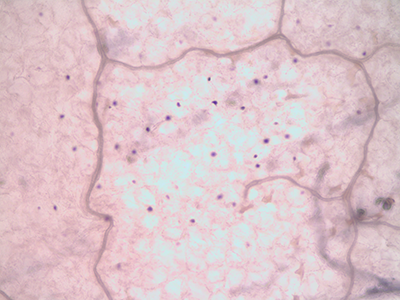

Supplement: S1 File — (ZIP) [file ppat.1010421.s001.zip › Fig 6 Images/WT 8dpi.tif]

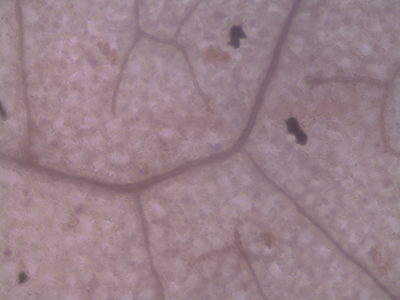

Supplement: S1 File — (ZIP) [file ppat.1010421.s001.zip › Fig 6 Images/Mock 12 dpi new.tif]

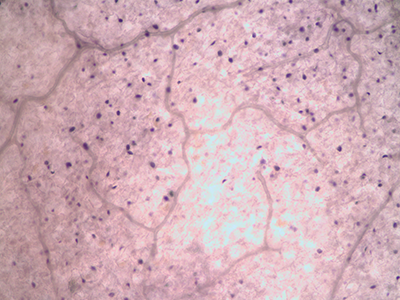

Supplement: S1 File — (ZIP) [file ppat.1010421.s001.zip › Fig 6 Images/WT 12dpi.tif]

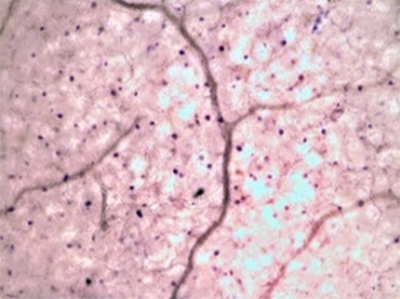

Supplement: S1 File — (ZIP) [file ppat.1010421.s001.zip › Fig 6 Images/U178G-U179G 12dpi.tif]

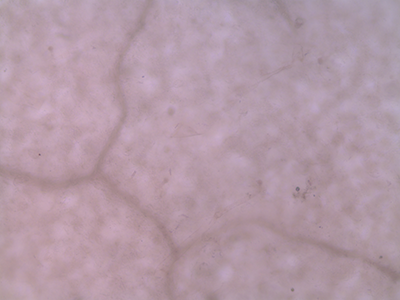

Supplement: S1 File — (ZIP) [file ppat.1010421.s001.zip › Fig 6 Images/A127G-C273G 10dpi new.tif]

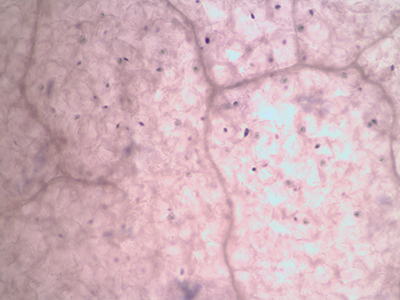

Supplement: S1 File — (ZIP) [file ppat.1010421.s001.zip › Fig 6 Images/U178G-U179G 10dpi.tif]

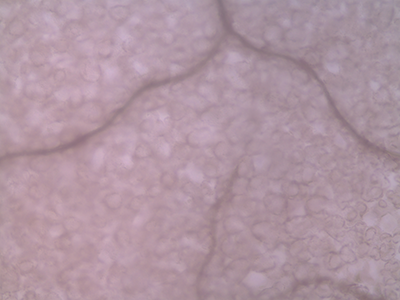

Supplement: S1 File — (ZIP) [file ppat.1010421.s001.zip › Fig 6 Images/Mock 10dpi new.tif]

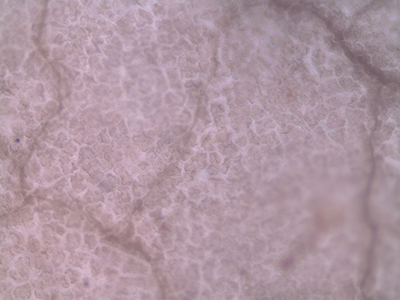

Supplement: S1 File — (ZIP) [file ppat.1010421.s001.zip › Fig 6 Images/Mock 8dpi new.tif]

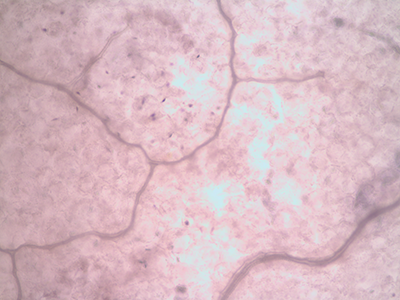

Supplement: S1 File — (ZIP) [file ppat.1010421.s001.zip › Fig 6 Images/U178G-U179G 8dpi.tif]

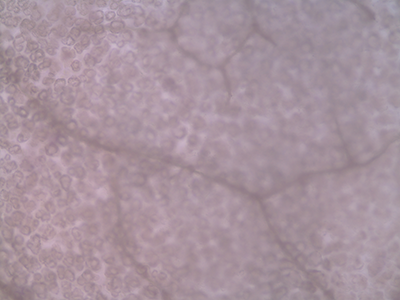

Supplement: S1 File — (ZIP) [file ppat.1010421.s001.zip › Fig 6 Images/A127G-C273G 12dpi new.tif]

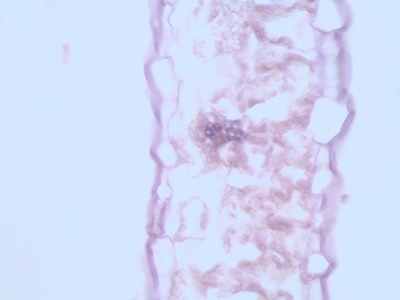

Supplement: S2 File — (ZIP) [file ppat.1010421.s002.zip › Fig 7 Images/Fig.7B new.tif]

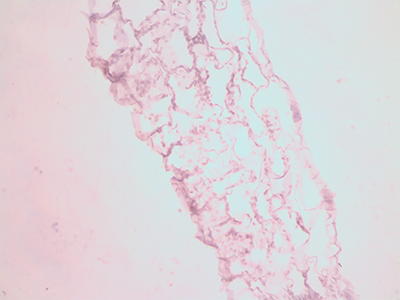

Supplement: S2 File — (ZIP) [file ppat.1010421.s002.zip › Fig 7 Images/Fig.7A new.tif]

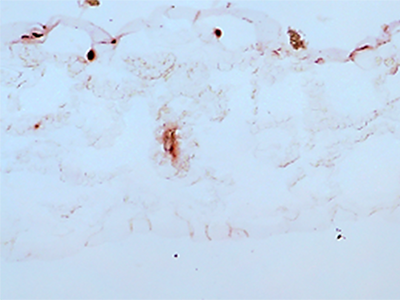

Supplement: S2 File — (ZIP) [file ppat.1010421.s002.zip › Fig 7 Images/Fig.7F.tif]

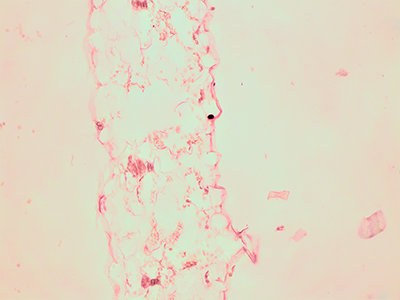

Supplement: S2 File — (ZIP) [file ppat.1010421.s002.zip › Fig 7 Images/Fig.7D.tif]

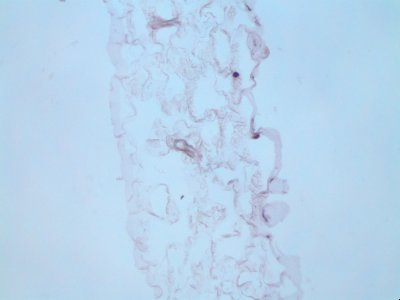

Supplement: S2 File — (ZIP) [file ppat.1010421.s002.zip › Fig 7 Images/Fig.7E.tif]

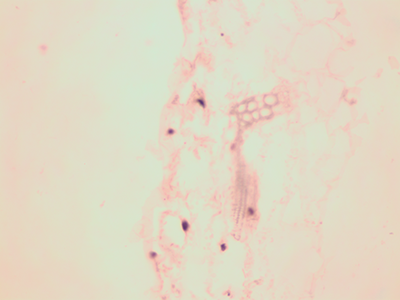

Supplement: S2 File — (ZIP) [file ppat.1010421.s002.zip › Fig 7 Images/Fig.7C.tif]

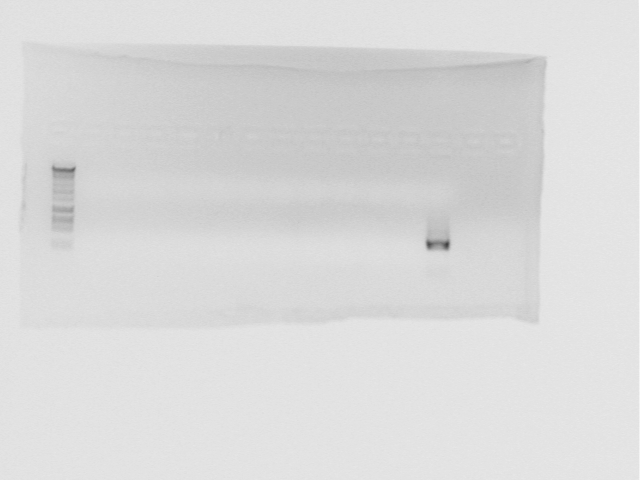

Supplement: S4 File — (ZIP) [file ppat.1010421.s004.zip › Fig 4 Images/Figure 4C U178G-U179G.tif]

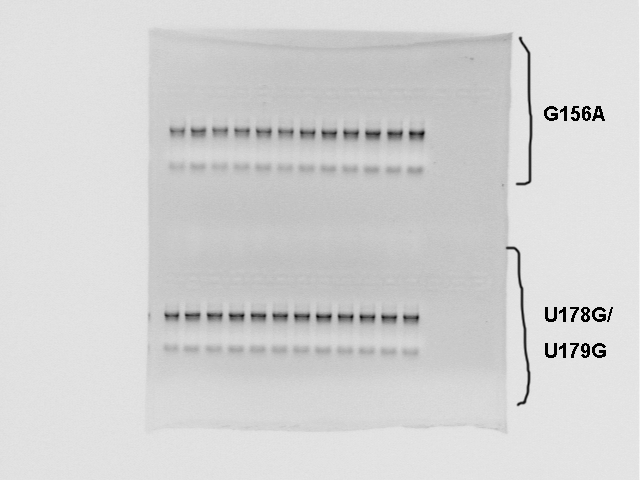

Supplement: S4 File — (ZIP) [file ppat.1010421.s004.zip › Fig 4 Images/Figure 4C actin.tif]

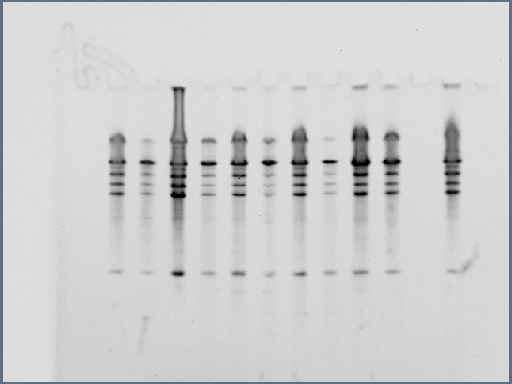

Supplement: S5 File — (ZIP) [file ppat.1010421.s005.zip › Fig 8 Images/Figure 8E EB staining.tif]

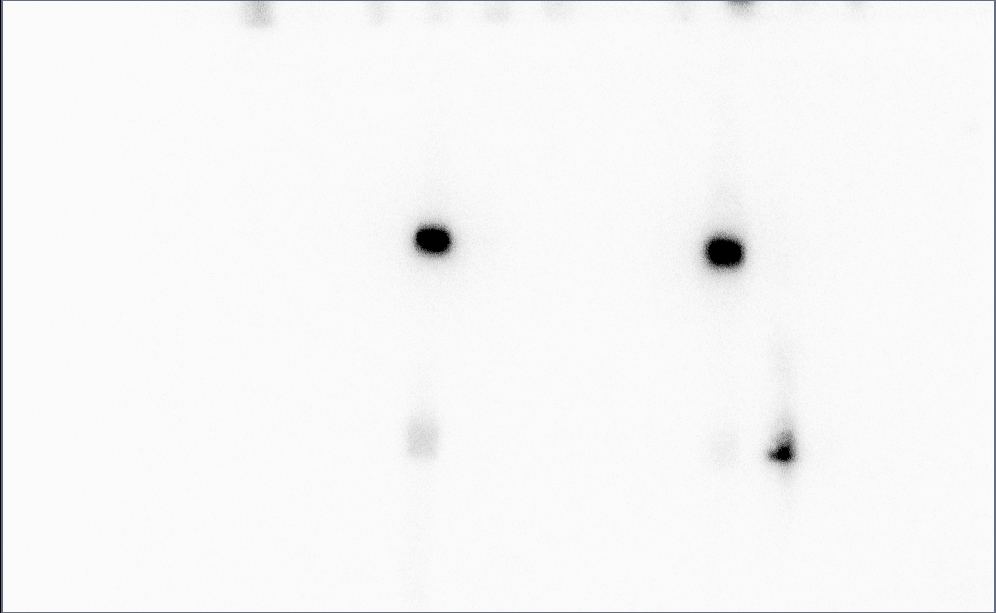

Supplement: S5 File — (ZIP) [file ppat.1010421.s005.zip › Fig 8 Images/Figure 8E P32.tif]

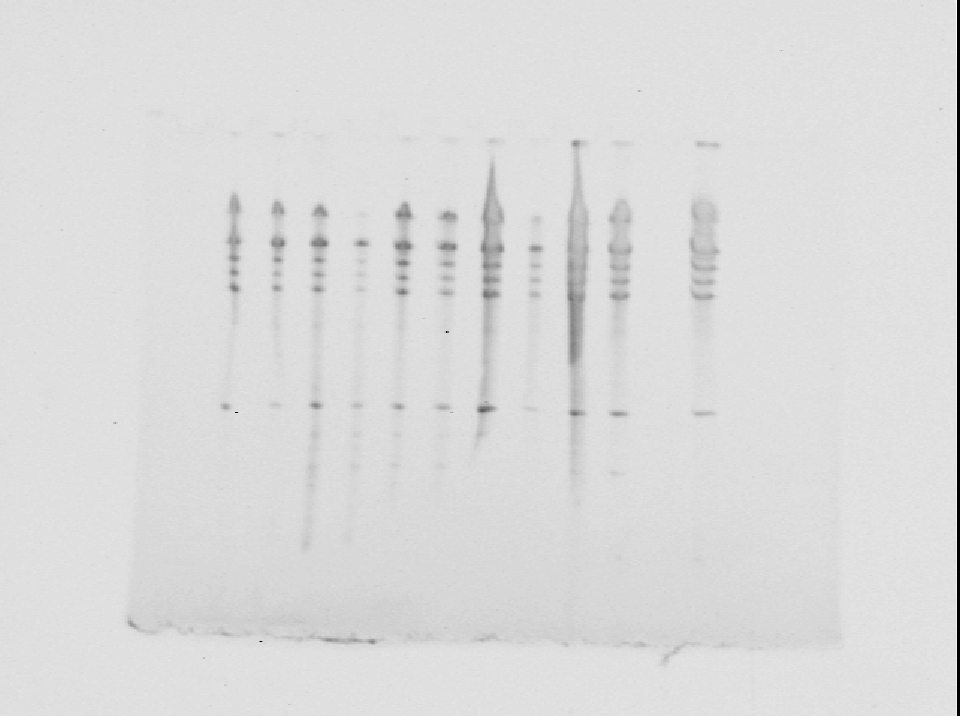

Supplement: S5 File — (ZIP) [file ppat.1010421.s005.zip › Fig 8 Images/Figure 8F EB staining.tif]

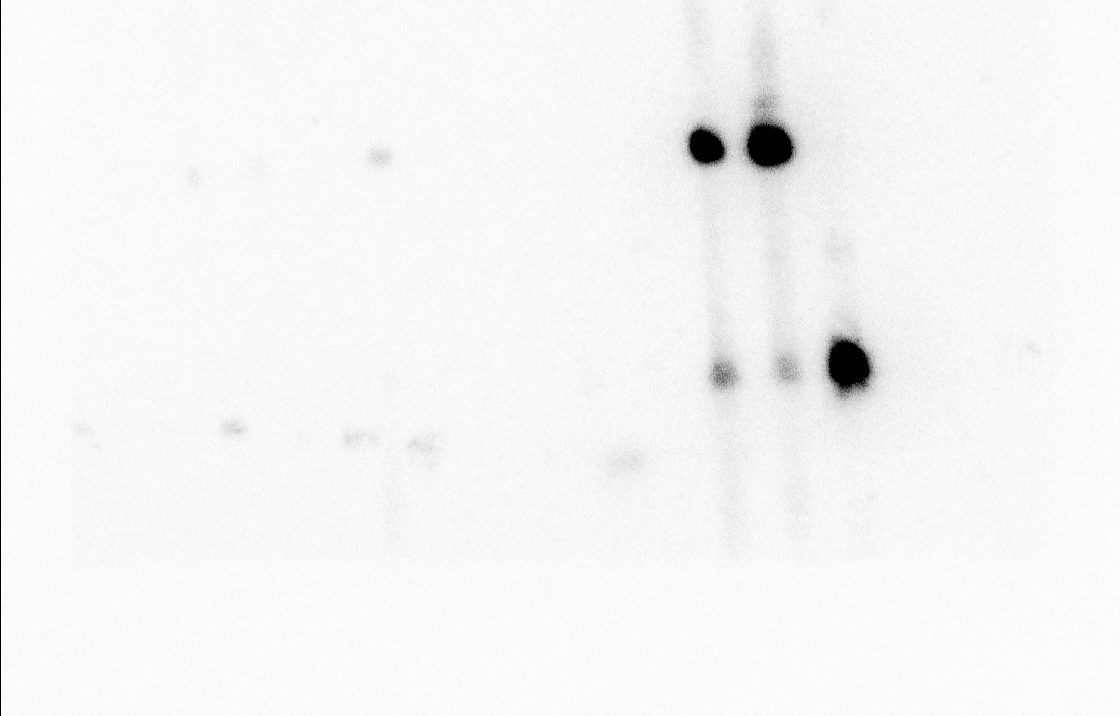

Supplement: S5 File — (ZIP) [file ppat.1010421.s005.zip › Fig 8 Images/Figure 8F P32.tif]
