# Supplementary material for: Correction: A three-dimensional RNA motif mediates directional trafficking of Potato spindle tuber viroid from epidermal to palisade mesophyll cells in Nicotiana benthamiana
Source: PLoS Pathog. 2022 Mar 22;18(3):e1010421. doi: 10.1371/journal.ppat.1010421 (PMC8939776; doi:10.1371/journal.ppat.1010421)
Supplement: S3 File — (ZIP) [file ppat.1010421.s003.zip › Fig. 6 and 7 Quantitative data/Fig.6B data.pdf]

Fig. 6B. Positive hybridzation signals/image field

8 dpi		10 dpi		12 dpi	
WT	178G-U179G	WT	178G-U179G	WT	178G-U179G
101	61	184	125	318	50
103	100	179	114	324	115
125	52	138	54	215	183
79	50	120	36	247	83
86	59	127	147	162	93
65	57	219	85	274	95
132	34	161	33	101	125
122	43	228	96	131	208
101	52	193	56	94	53
115	98	161	143	234	125
122	27	152	83	280	88
96	34	202	42	326	48
139	73	173	71	247	53
132	34	170	91	232	60
82	20	232	76	96	80
101	34	235	56	517	215
108	30	159	145	552	140
156	52	150	125	401	200
86	48	186	103	171	168
86	34	175	169	261	265
108	39	150	123	221	135
156	50	129	78	169	98
86	39	202	145	366	190
86	73	104	83	160	83
108	32	175	58	160	113
156	48	202	120	164	190
58	50	198	80	166	68
84	64	104	123	215	168
108	73	173	169	197	134
89	41	225	76	388	144
106	32	78	118	484	88
113	75	152	47	460	177
82	41	78	60	221	73
46	59	129	76	120	125
55	39	202	123	195	110
103	50	104	169	175	135
125	39	175	76	230	83
79	73	202	118	263	68
58	32	198	47	276	138
115	48	175	51	241	80
100	59	181	128	312	51
102	98	176	116	318	116
124	50	135	50	218	184
78	48	117	30	248	84
85	57	124	153	170	94

64	55	216	84	272	96
131	32	158	28	115	126
121	41	225	96	142	209
100	50	190	52	109	54
114	96	158	148	236	126
121	25	149	82	278	89
95	32	199	38	320	49
138	71	170	69	248	54
131	32	167	92	234	61
81	18	229	74	111	81
100	32	232	52	493	216
107	28	156	150	525	141
155	50	147	128	387	201
85	46	183	104	178	169
85	32	172	177	260	266
107	37	147	126	224	136
155	48	126	77	176	99
85	37	199	150	355	191
85	71	101	82	168	84
107	30	172	55	168	114
155	46	199	123	172	191
57	48	195	79	174	69
83	62	101	126	218	169
107	71	170	177	202	135
88	39	222	74	375	145
105	30	75	121	463	89
112	73	149	43	441	178
81	39	75	57	224	74
45	57	126	74	133	126
54	37	199	126	200	111
102	48	101	177	182	136
124	37	172	74	232	84
78	71	199	121	262	69
57	30	195	43	274	139
114	46	172	47	242	81
104	62	187	182	317	49
106	101	182	146	323	114
128	53	141	110	214	182
82	51	123	131	246	82
89	60	130	158	161	92
68	58	222	110	273	94
135	35	164	71	100	124
125	44	231	41	130	207
104	53	196	62	93	52
118	99	164	98	233	124
125	28	155	197	279	87
99	35	205	206	325	47
142	74	176	104	246	52

135	35	173	71	231	59
85	21	235	137	95	79
104	35	238	167	516	214
111	31	162	65	551	139
159	53	153	71	400	199
89	49	189	80	170	167
89	35	178	104	260	264
111	40	153	170	220	134
159	51	132	230	168	97
89	40	205	104	365	189
89	74	107	77	159	82
111	33	178	59	159	112
159	49	205	89	163	189
61	51	201	83	165	67
87	65	107	137	214	167
111	74	176	197	196	133
92	42	228	230	387	143
109	33	81	113	483	87
116	76	155	104	459	176
85	42	81	170	220	72
49	60	132	221	119	124
58	40	205	230	194	109
106	51	107	104	174	134
128	40	178	77	229	82
82	74	205	59	262	67
61	33	201	89	275	137
118	49	178	83	240	79
103	60	184	176	311	50
105	99	179	143	317	115
127	51	138	110	217	183
81	49	120	129	247	83
88	58	127	154	169	93
67	56	219	110	271	95
134	33	161	75	114	125
124	42	228	47	141	208
103	51	193	67	108	53
117	97	161	99	235	125
124	26	152	189	277	88
98	33	202	197	319	48
141	72	173	105	247	53
134	33	170	75	233	60
84	19	232	135	110	80
103	33	235	162	492	215
110	29	159	69	524	140
158	51	150	75	386	200
88	47	186	83	177	168
88	33	175	105	259	265
110	38	150	165	223	135

158	49	129	219	175	98
88	38	202	105	354	190
88	72	104	80	167	83
110	31	175	64	167	113
158	47	202	91	171	190
60	49	198	86	173	68
86	63	104	135	217	168
110	72	173	189	201	134
91	40	225	219	374	144
108	31	78	113	462	88
115	74	152	105	440	177
84	40	78	165	223	73
48	58	129	211	132	125
57	38	202	219	199	110
105	49	104	105	181	135
127	38	175	80	231	83
81	72	202	64	261	68
60	31	198	91	273	138
117	47	175	86	241	80
106	90	112	51	186	178
118	72	100	76	523	77
101	66	145	73	89	56
84	78	112	67	275	58
66	31	139	57	289	72
99	44	122	76	244	34
123	48	116	107	326	67
98	30	123	73	488	81
119	58	84	66	253	133
99	60	124	55	319	72
166	89	145	95	199	94
67	34	109	44	274	163
23	78	176	72	277	56
160	53	176	86	305	123
31	48	151	62	339	75
84	71	155	95	341	66
189	65	114	86	205	33
85	49	107	58	320	109
57	66	85	64	245	137
62	67	151	98	157	176
88	67	152	49	194	187
34	26	100	98	101	166
73	99	165	75	350	98
47	78	148	83	341	19
74	28	132	65	338	139
167	70	101	65	189	89
167	54	82	72	313	45
177	67	111	92	229	81
93	65	158	48	77	91

126	26	148	83	188	56
47	81	161	55	349	21
33	68	102	95	191	83
135	66	136	73	199	68
77	71	154	95	350	68
167	45	134	98	171	126
81	81	166	81	187	151
39	28	111	87	132	89
117	38	127	97	211	57
117	56	137	91	328	100
177	34	161	99	188	88
69	47	148	67	473	100
76	43	106	97	305	154
95	78	145	46	196	67
106	77	102	45	152	34
92	51	109	59	247	123
72	80	90	55	305	101
41	34	54	109	212	92
53	49	102	34	156	83
189	38	165	78	280	62
45	76	155	77	345	64
91	66	105	82	260	153
118	71	101	95	208	47
68	46	118	78	202	166
66	80	161	91	290	76
78	73	124	54	157	67
177	78	93	92	309	42
88	67	160	104	266	78
96	82	118	49	348	108
89	29	100	101	346	47
128	66	116	66	191	123
81	23	237	68	329	112
88	89	145	99	154	98
170	36	120	112	173	107
165	30	158	91	212	151
121	59	154	76	87	154
100		160	105	262	157
126		166	59	91	131
		106	92	210	137
		145	61	310	35
		142	65	274	77
		155	95	189	131
		183	89	89	131
		144	107	199	113
		113	88	224	122
		148	71	188	62
		118	72	294	64
		149	102	288	133

102	95	190	118
138	88	261	90
142	106	151	161
139	92	336	127
154	78	255	189
155	106	279	88
145	52	62	141
157	155	173	157
159	65	319	51
139	80	206	106
199	78	176	97
101	90	185	99
101	48	190	160
121	136	276	152
150	51	199	90
134	94	329	132
131	70	238	287
179	91	232	66
107	99	112	68
154	142	186	70
166	68	345	154
168	100	377	31
176	86	350	161
149	89	251	141
159	46	248	87
119	155	273	83
233	73	342	153
148	54	329	199
178	71	153	147
151	95	81	94
116	104	200	141
132	71	478	96
126	99	175	58
124	69	222	70
121	64	232	134
134	60	473	28
160	70	302	138
163	91	89	51
85	83	219	267
177	99	276	45
148	62	271	61
146	123	97	142
159	93	254	130
177	97	313	189
93	47	299	32
271	88	350	45
123	52	101	143
179	99	223	166

154	46	102	83
138	96	281	157
139	115	299	322
148	87	133	45
119	99	112	32
198	89	247	45
154	89	334	96
143	77	232	166
100	49	488	84
149	64	182	178
157	108	498	66
149	46	171	143
166	60	434	149
147	52	349	82
160	44	331	36
143	77	283	199
158	63	302	62
162	92	348	163
161	90	343	162
143	75	134	198
182	66	346	99
172	58	101	107
162	65	234	72
147	93	483	88
159	93	225	62
161	98	308	67
142	77		74
199	71		66
101	99		55
151	30		52
188	53		43
166	92		38
135	83		162
155	90		61
198	73		288
	92		61
	85		164
	92		42
	77		57
	87		45
	75		140
	73		87
	72		70
	73		139
	81		62
	96		141
	79		56
	93		45

94	61
91	148
78	56
88	143
95	154
96	356
72	121
75	87
82	86
77	71
77	136
93	140
77	144
93	151
81	79
89	145
81	137
97	68
88	60
76	152
76	75
97	88
94	189
90	41
72	79
79	43
93	147
86	94
74	109
83	198
99	87
88	137
86	129
78	61
99	78
90	147
94	57
95	106
84	18
77	157
78	103
85	63
71	58
94	78
97	167
95	104
89	183
85	54

93	158
76	119
78	108
88	23
79	23
73	87
95	151
86	21
93	68
75	61
86	158
89	147
81	89
94	144
81	72
99	11
76	152
84	131
94	298
77	66
84	68
97	164
83	78
90	143
74	278
82	62
89	312
71	64
81	109
84	84
72	68
78	67
72	70
73	141
75	143
84	138
75	35
95	55
77	57
122	163
85	77
92	187
91	37
78	166
98	52
90	85
84	149
83	73

75	178
86	34
93	67
83	16
98	86
83	16
94	66
88	36
135	64
87	77
127	198
81	18
71	34
80	189
73	31
85	59
82	93
96	56
144	134
98	159
84	66
175	
99	
78	
98	
76	
188	
74	
155	
81	
91	
86	
83	
122	
95	
75	
78	
76	
106	
78	
74	
98	
76	
98	
79	
91	
87	
86	

			82			
			88			
			75			
32	19	38	35	102	56	Standard Error
102	52	154	92	252	110	Average

Fig. 6B	U178G-U179G		error	WT	error
	8dpi	51.9	18.9	101.6	32.5
	10dpi	92.2	34.5	154.4	38.2
	12dpi	110.0	56.0	251.5	102.1
