# Supplementary material for: Correction: A three-dimensional RNA motif mediates directional trafficking of Potato spindle tuber viroid from epidermal to palisade mesophyll cells in Nicotiana benthamiana
Source: PLoS Pathog. 2022 Mar 22;18(3):e1010421. doi: 10.1371/journal.ppat.1010421 (PMC8939776; doi:10.1371/journal.ppat.1010421)
Supplement: S3 File — (ZIP) [file ppat.1010421.s003.zip › Fig. 6 and 7 Quantitative data/Fig.7G and H data.pdf]

Fig. 7. Positive Hybridization Signals/Section

			**Fig. 7G**	**12 dpi**			
WT	Sections	uEp	Pm	U178G/U179G	Sections	uEp	Pm
	1	1	4		1	2	0
	2	3	6		2	2	0
	3	1	6		3	1	0
	4	2	3		4	2	0
	5	3	5		5	0	0
	6	2	8		6	2	0
	7	4	7		7	0	0
	8	1	4		8	2	0
	9	3	5		9	1	0
	10	2	5		10	2	0
	11	3	6		11	1	0
	12	3	4		12	1	0
	13	2	5		13	2	0
	14	3	3		14	0	0
	15	1	8		15	1	0
	16	3	2		16	2	0
	17	3	7		17	0	0
	18	2	8		18	2	0
	19	1	6		19	1	0
	20	2	5		20	2	0
	21	2	4		21	1	0
	22	3	5		22	1	0
	23	1	5		23	2	0
	24	5	3		24	0	0
	25	4	6		25	2	0
	26	3	4		26	0	0
	27	1	2		27	2	0
	28	2	4		28	2	0
	29	2	4		29	1	0
	30	1	2		30	3	0
	31	2	1		31	2	0
	32	2	6		32	1	0
	33	3	7		33	2	0
	34	4	5		34	3	0
	35	1	5		35	1	0
	36	3	4		36	1	0
	37	3	3		37	2	0
	38	2	4		38	1	0
	39	1	3		39	1	0
	40	3	6		40	0	0
		93	190			54	0
	**Average**	2.325	4.75		**Average**	1.3	0
	**Standarc**	1.0096	1.6993		**Standarc**	0.781	0

			**Fig. 7H**	**18-20 dpi**			
WT	Sections	uEp	Pm	U178G/U179G	Sections	uEp	Pm
	1	3	7		1	1	0
	2	6	7		2	3	0
	3	4	8		3	2	0
	4	6	7		4	3	0
	5	2	5		5	3	1
	6	0	6		6	4	0
	7	4	2		7	3	0
	8	3	6		8	4	0
	9	2	8		9	4	0
	10	1	9		10	3	0
	11	4	6		11	3	0
	12	1	8		12	3	1
	13	2	9		13	2	0
	14	4	5		14	3	0
	15	3	8		15	1	0
	16	6	6		16	3	0
	17	0	5		17	2	0
	18	3	9		18	3	0
	19	4	5		19	3	0
	20	5	6		20	3	0
	21	6	5		21	2	0
	22	4	5		22	2	0
	23	4	3		23	3	0
	24	3	4		24	2	0
	25	3	4		25	3	0
	26	5	7		26	3	0
	27	3	5		27	1	0
	28	1	4		28	1	0
	29	5	4		29	2	0
	30	4	3		30	2	0
	31	3	6		31	1	0
	32	4	7		32	2	0
	33	3	5		33	3	0
	34	3	5		34	0	0
	35	4	3		35	0	0
	36	5	4		36	2	1
	37	4	6		37	1	0
	38	2	5		38	1	0
	39	2	4		39	1	0
	40	3	5		40	1	0
		134	226			89	3
	**Average**	3.35	5.65		**Average**	2.225	0.075
	**Standarc**	1.5256	1.74		**Standarc**	1.0365	0.2634

**Fig.7G**		WT	error bar	.78G/U17	error bar
	uEp	2.325	1.0096	1.3	0.781
	Pm	4.75	1.6993	0	0

**Fig.7H**		WT	error bar	.78G/U17	error bar
	uEp	3.35	1.52561463	2.225	1.0365
	Pm	5.65	1.739971264	0.075	0.2634
